# Supplementary material for: Multitrait genome-wide analyses identify new susceptibility loci and candidate drugs to primary sclerosing cholangitis
Source: Nat Commun. 2023 Feb 24;14:1069. doi: 10.1038/s41467-023-36678-8 (PMC9958016; doi:10.1038/s41467-023-36678-8)
Supplement: Supplementary file 1 — Supplementary Information [file 41467_2023_36678_MOESM1_ESM.pdf]

Multi-trait genome-wide joint analyses identify new susceptibility loci and candidate drugs to primary sclerosing cholangitis

Supplementary Information

Younghun Han[#], Jinyoung Byun[#], Catherine Zhu, Ryan Sun, Julia Y. Roh, Heather J. Cordell, Hyun-Sung Lee, Vikram R. Shaw, Sung Wook Kang, Javad Razjouyan, Matthew A. Cooley, Manal M. Hassan, Katherine A. Siminovitch, Trine Folseraas, David Ellinghaus, Annika Bergquist, Simon M Rushbrook, Andre Franke, Tom H Karlsen, Konstantinos N. Lazaridis, The International PSC Study Group[‡], Katherine A. McGlynn, Lewis R. Roberts, Christopher I. Amos*

[#]These authors contributed equally to this study.

[‡]The IPSCSG banner members and affiliations are listed in the Supplementary Information.

*Correspondence to

Christopher I. Amos, Institute for Clinical and Translational Research, Baylor College of Medicine, One Baylor Plaza, Houston, Texas, 77030; Phone: 713-798-3394; FAX: 713-798-1499; email: Chris.Amos@bcm.edu

GWAS summary statistics for PSC and other polygenic traits used in the present study.

We downloaded publicly available GWAS summary statistics from existing data resources, the NHGRI-EBI GWAS Catalog[1], the MR IEU OpenGWAS data[2,3], and the international PSC Study Group[4]. PSC summary statistics can be downloaded from the international PSC Study Group (IPSCSG; https://www.ipscsg.org/published-studies/) and the NHGRI-EBI GWAS Catalog (http://ftp.ebi.ac.uk/pub/databases/gwas/summary_statistics/GCST004001-GCST005000/GCST004030). The details, including data code, category of trait, sample size, number of SNPs, reference with PMID, and download link, are shown in Supplementary Data 1.

Estimates of genetic heritability and pairwise genetic correlation using LDSR analysis.

We implemented LD score regression (LDSR; ldsc v1.0.1, https://github.com/bulik/ldsc)[5,6] to estimate genome-wide SNP-array heritability (h2) representing the proportion of phenotypic variance explained by all common SNPs and to examine the shared genetic contribution (rg) of PSC against numerous polygenic traits using publicly available GWAS summary statistics and linkage disequilibrium (LD) information with European samples from 1000 Genome Project as a reference for the pattern of genome-wide LD. LDSR is a method regressing χ^2 statistics from summary statistics on LD scores, estimating genetic correlation without bias due to population stratification or cryptic relatedness[5,6]. By regressing SNP-level associations for two traits, (i.e., the product of Z scores, $Z_{\mathbf{PSC}} \times Z_{\mathbf{trait1}}$) and weighting each SNP by its LD score, which is an estimate of the total amount of genetic variations tagged by each variant, we can estimate the magnitude and direction of the shared genomic architecture between PSC and a tested trait. We first implemented the command option of LD Score with "munge_sumstats.py" to generate the ".sumstats" format from the summary statistics after selecting approximately 1.22M HapMap3 SNPs with minor allele frequency (MAF) > 0.01 and exclusion of multi-allelic SNPs and the major histocompatibility complex (MHC) region (Chr6:25Mb-34Mb) as recommended. The major histocompatibility complex (MHC) region was excluded from summary statistics because of the complex and extended LD pattern and genetic architecture of the MHC region[7-10]. We then applied "ldsc.py --rg PSC.sumstats.gz, trait1.sumstats.gz --ref-ld-chr eur_w_ld_chr/ --w-ld-chr eur_w_ld_chr/ --out PSC_trait1".

Joint association analysis of multi-traits on PSC.

We carried out MTAG[11] (mtag v1.0.8; https://github.com/JonJala/mtag) to discover new independent PSC risk-associated loci against polygenic traits that demonstrated strong genetic correlation with PSC through LDSR analysis. MTAG[11] estimates the effect size per SNP for each trait by incorporating information included in other correlated traits by utilizing

bivariate LD score regression to gauge sample overlaps between summary statistics from multiple GWAS as input in MTAG (since MTAG does not require summary statistics from independent samples[11] among the multiple GWAS). It then carries out a combined LD score regression and meta-analysis approach from the genetically related traits. MTAG aligned all alleles in all summary statistics analyzed, and SNPs not present in any summary statistics were removed further. The output from MTAG provides re-estimated effect size and P-value (P) per each SNP for each trait.

Summary statistics imputation.

Since MTAG utilizes a common set of SNPs that overlap among all tested traits, MTAG implementation with high-powered GWAS is recommended[11]. The number of SNPs for PBC is approximately 5M, and those for other immune-mediated disorders are approximately ~8M (PSC and lupus) and ~10M (IBD, CD, and UC). We imputed the GWAS summary statistics for PBC[12] using SSimp package[13] (ssimp v.0.5.6; https://github.com/zkutalik/ssimp_software) and European individuals from the 1000 Genomes Project phase 3 reference panel, filtering out SNPs with a MAF ≤ 0.005. This imputation increased the number of SNPs from 5,054,572 to 8,412,578 for subsequent analyses, such as LDSR and MTAG, after removing SNPs with poor imputation quality ($r^2 < 0.5$). We applied "ssimp --gwas in.chr22 \ --ref

~/reference_panels/1000genomes/ALL.chr22.phase3_shapeit2_mvncall_integrated_v5a.20130502.genotypes.vcf.gz \

--sample.names

~/reference_panels/1000genomes/integrated_call_samples_v3.20130502.ALL.panel/sample/super_ pop=EUR

--imputed.maf 0.005 --impute.range 22 --out out.chr22 " in SSimp as an example for chromosome 22 (as an example script for chromosome 22).

Colocalization between GWAS and Expression quantitative trait locus signals from the Genotype-Tissue Expression

Based on the prediction that risk variants may exert their effects via various tissues, we surveyed all 49 tissue types available in Genotype-Tissue Expression (GTEx)[14] v8. The sample size of each tissue is described in Supplementary Data 3. All variant-gene cis-expression quantitative trait locus (eQTL) associations tested in each tissue within ±100kb windows of the lead variant presented in MTAG_PSC were extracted. Colocalization of the MTAG_PSC and eQTL signals was calculated using the LD-independent colocalization approach (coloc v5.1.0; https://cran.r-project.org/web/packages/coloc/)[15]. Although the PSC GWAS[4] implemented population stratification analysis and focused on European-descent individuals, we applied the LD-independent approach to avoid spurious colocalizations due to the violation of common LD assumption

in the GWAS summary-level data. We considered the colocalizations when coloc suggested the plausible posterior probability that both PSC and a tissue from GTEx v8 are associated and share a single function variant (PP4 > 0.80).

Gene-based functional enrichment analysis using STRING database and DAVID Bioinformatics Resources

We carried out gene-based enrichment analysis of protein-protein interaction (PPI) networks amongst 406 potential candidate genes from position mapping, eQTL mapping, and chromatin interaction mapping as well as newly 19 MTAG-identified and previously reported PSC risk-associated genes using STRING PPI networks (STRING v11.5; https://string-db.org/cgi/input?sessionId=bmwWOuutn8ZR). We selected the setting options with the max number of interactions = 20 and the highest confidence score of 0.9 to survey the functional enrichment of numerous pathways by genes. We also utilized the Database for Annotation, Visualization, and Integrated Discovery (DAVID v6.8; https://david.ncifcrf.gov/) Bioinformatics Resources to survey the enrichment of various functional annotations (count > 2, P-value < 0.05).

Network-based proximity between drugs and disease-identified proteins for drug repurposing

To estimate a drug-disease proximity measures, distance (*d*) and the corresponding relative proximity (*z*), we implemented network-based proximity analysis for drug repurposing developed by Guney et al.[16]. The method provides a relative measure that quantifies the network-based proximity (or closeness) between drugs and disease proteins encoded by genes associated with disease[12,16]. We obtained the drug and drug target data from the DrugBank database[17] (version 5.1.9, https://go.drugbank.com/releases/latest, accessed in April 2022). In this study, we selected 39 PSC-associated genes from previously reported genome-wide significant associations[18] and newly MTAG-identified associations at $P=5.00\times10^{-8}$ (Supplementary Data 16). Among them, 32 candidate genes were converted to NCBI gene IDs using Ensembl BioMart[19]and used to calculate the relative proximity between each drug in the DrugBank database (v5.1.9) and a set of candidate proteins for PSC (Supplementary Table 1). All required packages are available at the emreg00 GitHub repository (https://github.com/emreg00)[16].

Supplementary References

1. Buniello A, MacArthur JAL, Cerezo M, Harris LW, Hayhurst J, Malangone C, et al. The NHGRI-EBI GWAS Catalog of published genome-wide association studies, targeted arrays and summary statistics 2019. Nucleic Acids Res. 2019;47(D1):D1005-d12.
2. Elsworth B, Lyon M, Alexander T, Liu Y, Matthews P, Hallett J, et al. The MRC IEU OpenGWAS data infrastructure. bioRxiv. 2020:2020.08.10.244293.
3. Lyon M, Andrews SJ, Elsworth B, Gaunt TR, Hemani G, Marcora E. The variant call format provides efficient and robust storage of GWAS summary statistics. bioRxiv. 2020:2020.05.29.115824.
4. Ji SG, Juran BD, Mucha S, Folseraas T, Jostins L, Melum E, et al. Genome-wide association study of primary sclerosing cholangitis identifies new risk loci and quantifies the genetic relationship with inflammatory bowel disease. Nat Genet. 2017;49(2):269-73.

5. Bulik-Sullivan B, Finucane HK, Anttila V, Gusev A, Day FR, Loh PR, et al. An atlas of genetic correlations across human diseases and traits. Nat Genet. 2015;47(11):1236-41.
6. Bulik-Sullivan BK, Loh PR, Finucane HK, Ripke S, Yang J, Schizophrenia Working Group of the Psychiatric Genomics C, et al. LD Score regression distinguishes confounding from polygenicity in genome-wide association studies. Nat Genet. 2015;47(3):291-5.
7. Byun J, Han Y, Ostrom QT, Edelson J, Walsh KM, Pettit RW, et al. The Shared Genetic Architectures Between Lung Cancer and Multiple Polygenic Phenotypes in Genome-Wide Association Studies. Cancer Epidemiol Biomarkers Prev. 2021;30(6):1156-64.
8. Pettit RW, Byun J, Han Y, Ostrom QT, Edelson J, Walsh KM, et al. The shared genetic architecture between epidemiological and behavioral traits with lung cancer. Sci Rep. 2021;11(1):17559.
9. Ostrom QT, Edelson J, Byun J, Han Y, Kinnersley B, Melin B, et al. Partitioned glioma heritability shows subtype-specific enrichment in immune cells. Neuro Oncol. 2021.
10. Byun J, Han Y, Walsh KM, Park AS, Bondy ML, Amos CI. Shared genomic architecture between COVID-19 severity and numerous clinical and physiologic parameters revealed by LD score regression analysis. Sci Rep. 2022;12(1):1891.
11. Turley P, Walters RK, Maghzian O, Okbay A, Lee JJ, Fontana MA, et al. Multi-trait analysis of genome-wide association summary statistics using MTAG. Nat Genet. 2018;50(2):229-37.
12. Cordell HJ, Fryett JJ, Ueno K, Darlay R, Aiba Y, Hitomi Y, et al. An international genome-wide meta-analysis of primary biliary cholangitis: Novel risk loci and candidate drugs. J Hepatol. 2021;75(3):572-81.
13. Rueger S, McDaid A, Kutalik Z. Evaluation and application of summary statistic imputation to discover new height-associated loci. PLoS Genet. 2018;14(5):e1007371.
14. Consortium G. The Genotype-Tissue Expression (GTEx) project. Nat Genet. 2013;45(6):580-5.
15. Wallace C. Eliciting priors and relaxing the single causal variant assumption in colocalisation analyses. PLoS Genet. 2020;16(4):e1008720.
16. Guney E, Menche J, Vidal M, Barabasi AL. Network-based in silico drug efficacy screening. Nat Commun. 2016;7:10331.
17. Wishart DS, Feunang YD, Guo AC, Lo EJ, Marcu A, Grant JR, et al. DrugBank 5.0: a major update to the DrugBank database for 2018. Nucleic Acids Res. 2018;46(D1):D1074-D82.
18. MacArthur J, Bowler E, Cerezo M, Gil L, Hall P, Hastings E, et al. The new NHGRI-EBI Catalog of published genome-wide association studies (GWAS Catalog). Nucleic Acids Res. 2017;45(D1):D896-D901.
19. Cunningham F, Allen JE, Allen J, Alvarez-Jarreta J, Amode MR, Armean IM, et al. Ensembl 2022. Nucleic Acids Res. 2022;50(D1):D988-D95.

A full list of members of the International PSC Study Group (IPSCSG) banner.

Christoph Schramm[24], David Shapiro[25], Elizabeth Goode[26], Kelly Spiess[26], Brijesh Srivastava[26], George Mells[26], Richard N Sandford[26], Olivier Chazouillères[27], Sven Almer[28], Pietro Invernizzi[29], Ana Lleo[29], Marco Carbone[29], Erik M Schlicht[30], Aliya F Gulamhusein[30], Brian D Juran[30], John E Eaton[30], Hugh E. Mulcahy[31], David H. Adams[32], Gideon M Hirschfield[32], Alexander Knuth[33], Mattias Laudes[34], Alastair Burt[35], Gregory Gores[36], Severine Vermeire[37], Ulrich Beuers[38], Henk R. van Buuren[39], Andrzej Habior[40], Andreas E. Kremer[38], Rinse K Weersma[41], Hiromasa Ohira[42], Shahid A Khan[43], Tim Lankisch[44], Michael P Manns[44], Tobias J Weismüller[44], Marco Marzioni[45], George Webster[46], Beate K. Straub[47], Peter Schirmacher[47], Felix Braun[48], Cisca Wijmenga[49], Martina Sterneck[50], Beat Müllhaup[51], Chantal Housset[52], Emina Halilbasic[53], Michael Trauner[53], Peter Sauer[54], Mario Strazzabosco[55], Einar Björnsson[56], Dieter Häussinger[57], Verena Keitel[57], Tobias Müller[58], Werner Van Steenbergen[59], Christian Rust[60], Andreas Teufel[61], Graeme Alexander[62], Joseph A Odin[63], Simon D. Taylor-Robinson[64], Atsushi Tanaka[65], Daniel N Gotthardt[66], Kimmo Kontula[67], Hanns-Ulrich Marschall[68], Saul J. Karpen[69], Luca Fabris[70], Annarosa Floreani[71], Roger W Chapman[72], Carlo Selmi[73], Fredrik Rorsman[74], Elizabeth J Atkinson[75], Mariza de Andrade[75], Peter Fickert[76], Keith Lindor[77], Andreas Geier[78], David Goldberg[79], Jayant Talwalkar[79], Yoshiyuki Ueno[80], Kapil B Chopra[81], Christopher L Bowlus[82], Lina Lindstrøm[83], Niklas Björkström[83], Velimir A Luketic[84], Kate Williamson[85], Cleo van Diemen[86], Pieter van der Vlies[86], Martti Farkkila[87], Ansgar Lohse[88], Naga Chalasani[89], David Jones[90], Stefan Schreiber[17], Sören Mucha[17], Eva Ellinghaus[17], Gunnar Jacobs[91], Wolfgang Lieb[91], Jennifer Sambrook[92], Johan Fevery[93], Schalk Van der Merwe[93], Piotr Milkiewicz[94], Kris V Kowdley[95], Catalina Coltescu[96], Albert Pares[97], Ewa Wunsch[98], Ruben Plentz[99], Dennis Black[100], Carmel Moore[101], David J Roberts[101], John Danesh[101], Willem H Ouwehand[101], Espen Melum[16], Kirsten M Boberg[16], LarsAabakken[16], Peter R Durie[102], Arthur Kaser[103], Christophe Corpechot[104], Raoul Poupon[104], Andrew Burroughs[105], Bertus Eksteen[106], Stephen Pereira[107], Daniel Burger[108], Frank G. Schaap[109], Serge Zweers[109], Hugh Harley[110], Luke Jostins[111], Daniel J Gaffney[112], Carl A Anderson[112], Javier Gutierrez-Achury[112], Jimmy Z Liu[112], Natsuhiko Kumasaka[112], Sun-Gou Ji[112], Tejas Shah[112], Trine Folseraas[16], David Ellinghaus[17], Annika Bergquist[18], Simon M Rushbrook[19,20], Andre Franke[17], Tom H Karlsen[21], Konstantinos N. Lazaridis[22]

Affiliations of the IPSCSG banner authors

24. 1st Department of Medicine, University Medical Center Hamburg-Eppendorf, Hamburg, Germany.
25. 4350 La Jolla Village Drive Suite 960, San Diego, CA 92122, USA.
26. Academic Department of Medical Genetics, University of Cambridge, Cambridge, UK.
27. AP-HP Hôpital Saint Antoine, Department of Hepatology, UPMC University Paris 6, Paris, France.
28. Avd. för Gastroenterologi och Hepatologi, Institutionen för Klinisk och Experimentell Medicin (IKE), The University Hospital, Linköping, Sweden.
29. Center for Autoimmune Liver Diseases, Humanitas Clinical and Research Center, Rozzano, Milan, Italy.
30. Center for Basic Research in Digestive Diseases, Division of Gastroenterology and Hepatology, Mayo Clinic College of Medicine, Rochester, Minnesota, USA.
31. Centre for Colorectal Diseases, St Vincent's University Hospital, Dublin, Ireland.
32. Centre for Liver Research, MRC Centre for Immune Regulation, Institute for Biomedical Research, University of Birmingham, Birmingham, B15 2TT, UK.
33. Clinic and Policlinic for Oncologie, University Hospital Zürich, Zürich, Switzerland.
34. Clinic of Internal Medicine I, University Hospital SchleswigHolstein, Kiel, Germany.
35. Clinical Deanery, Faculty of Medical Sciences, Newcastle University, Newcastle, UK.
36. College of Medicine, Mayo Clinic, Rochester, MN, USA.
37. Department of Clinical and Experimental Medicine, Katholieke Universiteit Leuven, Leuven, Belgium.
38. Department of Gastroenterology and Hepatology, Academic Medical Center, University of Amsterdam, Amsterdam, the Netherlands.
39. Department of Gastroenterology and Hepatology, Erasmus University Medical Centre, Rotterdam, The Netherlands.
40. Department of Gastroenterology, Hepatology and Clinical Oncology Centre of Postgraduate Medical Education, Warsaw, Poland.
41. Department of Gastroenterology and Hepatology, University of Groningen, University Medical Centre Groningen, Groningen, the Netherlands.
42. Department of Gastroenterology and Rheumatology, Fukushima Medical University School of Medicine, Fukushima, Japan.

43. Department of Gastroenterology, Hammersmith Hospital Campus, London, UK.
44. Department of Gastroenterology, Hepatology and Endocrinology, Hannover Medical School, Hannover, Germany.
45. Department of Gastroenterology, Università Politecnica delle Marche, Ospedali Riuniti University Hospital, Ancona, Italy.
46. Department of Gastroenterology, University College Hospital, London, UK.
47. Department of General Pathology, Institute of Pathology, Heidelberg, Germany.
48. Department of General, Visceral, Thoracic, Transplantation and Pediatric Surgery, University Medical Centre Schleswig-Holstein, Campus Kiel, Kiel, Germany.
49. Department of Genetics, University of Groningen, University Medical Centre Groningen, Groningen, the Netherlands.
50. Department of Hepatobiliary Surgery and Transplantation, University Medical Center Hamburg-Eppendorf, Hamburg, Germany.
51. Department of Hepatology and Gastroenterology, Swiss Hepato-Pancreatico-Biliary (HPB) and Transplant Center, University Hospital Zurich, Zurich, Switzerland.
52. Department of Hepatology, Reference Center for Inflammatory Biliary Diseases and Autoimmune Hepatitis (CRMR MIVB-H, ERN RARE-LIVER), Assistance Publique-Hôpitaux de Paris (AP-HP), Saint-Antoine Hospital, Paris, France.
53. Department of Internal Medicine III, Divison of Gastroenterology and Hepatology Medical University of Vienna Waehringer, Vienna, Austria.
54. Department of Internal Medicine IV, University Hospital of Heidelberg, Heidelberg, Germany.
55. Department of Internal Medicine Section of Digestive Diseases Yale University, New Haven, CT, USA.
56. Department of Internal Medicine, Division of Gastroenterology and Hepatology, Landspitali University Hospital, Reykjavik, Iceland.
57. Department of Internal Medicine, Gastroenterology, Hepatology and Infectiology, Heinrich Heine Universität, Düsseldorf, Germany.
58. Department of Internal Medicine, Hepatology and Gastroenterology, Charité Universitätsmedizin Berlin, Berlin, Germany.
59. Department of Liver, Biliary and Pancreatic Diseases, University Hospital Gasthuisberg, Catholic University of Leuven, Leuven, Belgium.
60. Department of Medicine 2, Grosshadern, University of Munich, Munich, Germany.
61. Department of Medicine I, University Medical Center, Regensburg, Germany.
62. Department of Medicine, Division of Hepatology, University of Cambridge, Cambridge.
63. Department of Medicine, Mount Sinai School of Medicine, New York, New York, USA.
64. Department of Medicine, St Mary's Hospital Campus, Imperial College London, UK.
65. Department of Medicine, Teikyo University School of Medicine, Tokyo, Japan.
66. Department of Medicine, University Hospital of Heidelberg, Heidelberg, Germany.
67. Department of Medicine, University of Helsinki, Helsinki, Finland.
68. Department of Molecular and Clinical Medicine, Institute of Medicine, Sahlgrenska Academy, University of Gothenburg, Gothenburg, Sweden.
69. Department of Pediatrics, Baylor College of Medicine, Houston, Texas, USA.
70. Department of Surgical and Gastroenterological Sciences, University of Padua, Padua, Italy.
71. Department of Surgical, Oncological and Gastroenterological Sciences, University of Padova, Padova, Italy.
72. Department of Translational Gastroenterology, Oxford University Hospitals NHS Trust, Oxford, UK.
73. Department of Translational Medicine, University of Milan, IRCCS Istituto Clinico Humanitas, Milan, Italy.
74. Department of Gastroenterology and Hepatology, Uppsala Univeristy Hospital, Uppsala, Sweden.
75. Division of Biomedical Statistics and Informatics, Mayo Clinic, Rochester, Minnesota, USA.
76. Division of Gastroenterology and Hepatology, Dept. of Medicine, Medical University of Graz, Graz, Austria.
77. Division of Gastroenterology and Hepatology, Mayo Clinic, Rochester, MN, USA.
78. Division of Gastroenterology and Hepatology, University Hospital Zürich, Zürich, Switzerland.
79. Division of gastroenterology, Dept. of Medicine, Hospital of the University of Pennsylvania and Perelman Center for Advanced Medicine, USA.
80. Division of Gastroenterology, Graduate School of Medicine, Tohoku University, Sendai, Japan.
81. Division of Gastroenterology, Hepatology and Nutrition, University of Pittsburgh, Pittsburgh, Pennsylvania, USA.
82. Division of Gastroeterology and Hepatology, University of California at Davis, Davis, California, USA.
83. Gastrocentrum Medicin, Karolinska Univ, Stockholm, Sweden.
84. Gastroenterology and Hepatology Section, Virginia Commonwealth University, Richmond, Virginia, USA.

85. Gastroenterology Department, John Radcliffe Hospital, Oxford University Hospitals, Oxford, UK.
86. Genome Analysis Facility, Dept. of Genetics, University Medical Centre Groningen, Groningen, The Netherlands.
87. Helsinki University and Helsinki University Hospital, Clinic of Gastroenterology, Helsinki, Finland.
88. I. Medizinische Klinik und Poliklinik, Universitätsklinikum Hamburg-Eppendorf, Hamburg, Germany.
89. Indiana University School of Medicine, Indianapolis, Indiana, USA.
90. Institute of Cellular Medicine (Hepatology), The Medical School, Newcastle University, Newcastle upon Tyne, UK.
91. Institute of Epidemiology and Biobank PopGen, University Hospital Schleswig-Holstein, Kiel, Germany.
92. INTERVAL Coordinating Centre, Department of Public Health and Primary Care, University of Cambridge, Cambridge, UK.
93. Laboratory of Hepatology, University Hospital Gasthuisberg, Leuven, Belgium.
94. Liver and Internal Medicine Unit, Department of General, Transplant and Liver Surgery, Medical University of Warsaw, Warsaw, Poland.
95. Liver Care Network and Organ Care Research, Swedish Medical Center, Seattle, Washington, USA.
96. Liver Centre, Toronto Western Hospital, Toronto, Ontario, Canada.
97. Liver Unit, Hospital Clínic, IDIBAPS, CIBERehd, University of Barcelona, Barcelona, Spain.
98. Liver Unit, Pomeranian Medical University, Szczecin, Poland.
99. Medizinische Universitätsklinik, Abteilung Innere Medizin 1, Tübingen, Germany.
100. Memphis Medical Center, Memphis, Tennessee, USA.
101. NIHR Blood and Transplant Research Unit in Donor Health and Genomics, Department of Public Health and Primary Care, University of Cambridge, Cambridge, UK.
102. Physiology and Experimental Medicine, Research Institute, Hospital for Sick Children, Toronto, Ontario, Canada.
103. Section of Gastroenterology and Hepatology Medical Department, Addenbrooke's Hospital, Cambridge, UK.
104. Service d'Hépatologie, Centre de référence des Maladies Inflammatoires des Voies Biliaires, Hôpital Saint-Antoine and UMR_S938, Faculté de Médecine Saint-Antoine, Université Pierre et Marie Curie (UPMC), Paris, France.
105. Sheila Sherlock Liver Centre and Department of Surgery, Royal Free Hospital, London, UK.
106. Snyder Institute for Chronic Diseases, Department of Medicine, University of Calgary, Calgary, Alberta, Canada.
107. The UCL Institute of Hepatology, Royal Free Hospital, London, UK.
108. Translational Gastroenterology Unit, John Radcliffe Hospital, Oxford Radcliffe NHS Trust, Oxford, UK.
109. Tytgat Institute for Liver and Intestinal research, Academic Medical Center, Amsterdam, The Netherlands.
110. University of Adelaide, Head of Clinical Hepatology, Co-Director of Viral Hepatitis Centre, Royal Adelaide Hospital, Australia.
111. Wellcome Trust Centre for Human Genetics, University of Oxford, Oxford, UK.
112. Wellcome Trust Sanger Institute, Wellcome Trust Genome Campus, Hinxton, UK.

Supplementary Table 1. PSC risk-associated candidate protein input for drug repurposing analysis. Candidate proteins were selected based on a genome-wide significant P-value shown in Supplementary Data 16.

Gene stable ID version	Gene name	NCBI gene ID	Gene stable ID version	Gene name	NCBI gene ID
ENSG00000178562.13	CD28	940	ENSG00000038532.10	CLEC16A	23274
ENSG00000178623.7	GPR35	2859	ENSG00000105287.8	PRKD2	25865
ENSG00000109471.4	IL2	3558	ENSG00000114861.14	FOXP1	27086
ENSG00000134460.11	IL2RA	3559	ENSG00000198218.6	QRICH1	54870
ENSG00000068383.14	INPP5A	3632	ENSG00000061273.13	HDAC7	51564
ENSG00000128604.14	IRF5	3663	ENSG00000160185.9	UBASH3A	53347
ENSG00000109323.4	MANBA	4126	ENSG00000138684.3	IL21	59067
ENSG00000173531.11	MST1	4485	ENSG00000112182.10	BACH2	60468
ENSG00000204842.10	ATXN2	6311	ENSG00000142606.11	MMEL1	79258
ENSG00000196628.9	TCF4	6925	ENSG00000103047.3	TANGO6	79613
ENSG00000118503.10	TNFAIP3	7128	ENSG00000124780.9	KCNK17	89822
ENSG00000183527.7	PSMG1	8624	ENSG00000167037.14	SGSM1	129049
ENSG00000153094.17	BCL2L11	10018	ENSG00000162927.9	PUS10	150962
ENSG00000111252.6	SH2B3	10019	ENSG00000119919.9	NKX2-3	159296
ENSG00000150637.4	CD226	10666	ENSG00000168071.17	CCDC88B	283234
ENSG00000170145.4	SIK2	23235	ENSG00000266760.1	MIR4464	100616109

Supplementary Figure 1. LocusZoom plots of regional genome-wide associations for MTAG-identified PSC-specific new susceptibility associations. LocusZoom genomic regional plot displays a new PSC-specific risk locus identified from the multi-trait analysis of GWAS (MTAG) in the discovery study. All presented P-values (p-value) are two-sided unadjusted P-values. The newly MTAG-identified PSC risk variant is colored in purple, and colors dots indicate LD measure r2 with the lead variant in purple**. (a) - (g)** displays newly discovered loci in this study and **(h)** new lead variant in the previously reported locus. **(a)** rs7608697 in ***PUS10*** on 2p16.1; **(b)** rs228614 in ***MANBA*** on 4q24; **(c)** rs12198665 in ***KCNK17*** on 6p21.2; **(d)** rs17780429 in ***TNFAIP3*** on 6q23.3; **(e)** rs3757387 in ***IRF5*** on 7q32.1; **(f)** rs791168 in ***NKX2-3*** on 10q24.2; **(g)** rs79390277 in ***TANGO6*** on 16q22.1; **(h)** rs6787808 in ***QRICH1*** on 3p21.31. LocusZoom (v1.4; https://github.com/statgen/locuszoom-standalone) was used to visualize regional GWAS results.

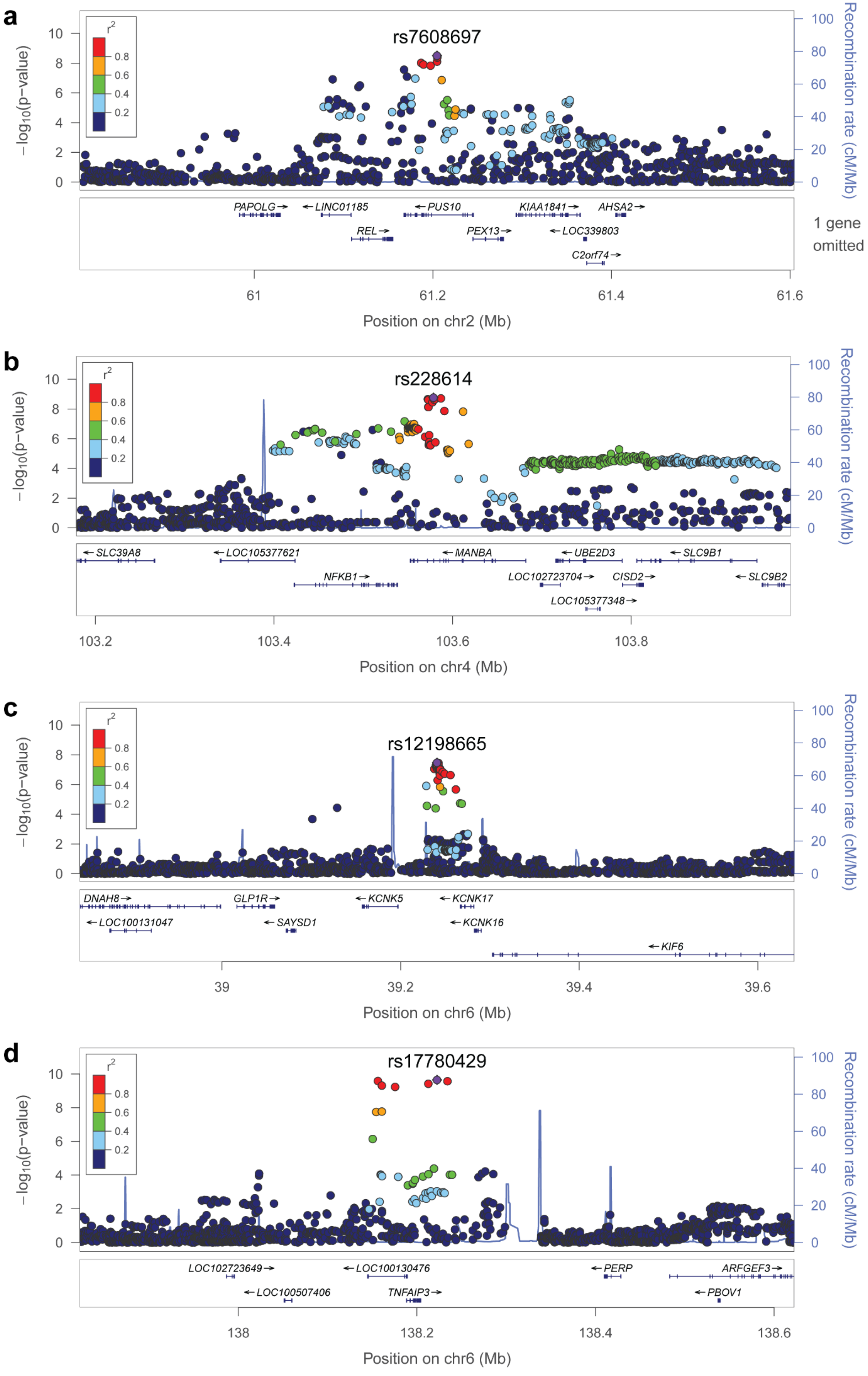

a
rs7608697
−log10(p−value)
Recombination rate (cM/Mb)
r2
0.8
0.6
0.4
0.2
PAPOLG→
←LINC01185
←PUS10
KIAA1841→
AHSA2→
REL→
PEX13→
←LOC339803
C2orf74→
1 gene omitted
61
61.2
61.4
61.6
Position on chr2 (Mb)
b
rs228614
−log10(p−value)
Recombination rate (cM/Mb)
←SLC39A8
←LOC105377621
←MANBA
←UBE2D3
←SLC9B1
NFKB1→
LOC102723704→
CISD2→
←SLC9B2
LOC105377348→
103.2
103.4
103.6
103.8
Position on chr4 (Mb)
c
rs12198665
−log10(p−value)
Recombination rate (cM/Mb)
DNAH8→
GLP1R→
←KCNK5
←KCNK17
←LOC100131047
←SAYSD1
←KCNK16
←KIF6
39
39.2
39.4
39.6
Position on chr6 (Mb)
d
rs17780429
−log10(p−value)
Recombination rate (cM/Mb)
LOC102723649→
←LOC100130476
←PERP
ARFGEF3→
←LOC100507406
TNFAIP3→
←PBOV1
138
138.2
138.4
138.6
Position on chr6 (Mb)

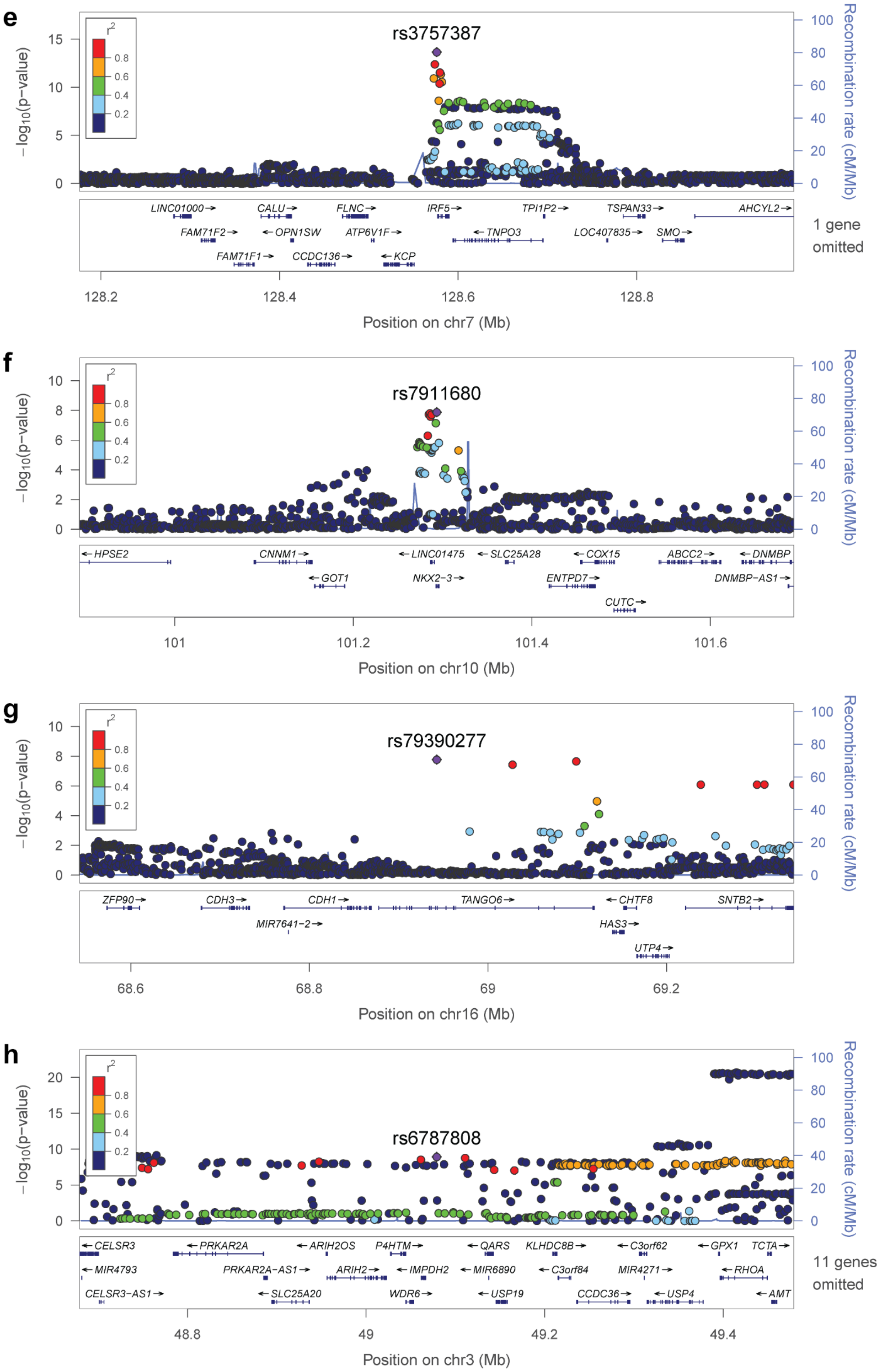
e
rs3757387
r2
0.8
0.6
0.4
0.2
−log10(p−value)
Recombination rate (cM/Mb)
LINC01000→
CALU→
FLNC→
IRF5→
TPI1P2→
TSPAN33→
AHCYL2→
FAM71F2→
←OPN1SW
ATP6V1F→
←TNPO3
LOC407835→
SMO→
FAM71F1→
CCDC136→
←KCP
1 gene omitted
128.2
128.4
128.6
128.8
Position on chr7 (Mb)
f
rs7911680
←HPSE2
CNNM1→
←LINC01475
←SLC25A28
←COX15
ABCC2→
←DNMBP
←GOT1
NKX2−3→
ENTPD7→
DNMBP−AS1→
CUTC→
101
101.2
101.4
101.6
Position on chr10 (Mb)
g
rs79390277
ZFP90→
CDH3→
CDH1→
TANGO6→
←CHTF8
SNTB2→
MIR7641−2→
HAS3→
UTP4→
68.6
68.8
69
69.2
Position on chr16 (Mb)
h
rs6787808
←CELSR3
←PRKAR2A
←ARIH2OS
P4HTM→
←QARS
KLHDC8B→
←C3orf62
←GPX1
TCTA→
←MIR4793
PRKAR2A−AS1→
ARIH2→
←IMPDH2
←MIR6890
←C3orf84
MIR4271→
←RHOA
CELSR3−AS1→
←SLC25A20
WDR6→
←USP19
CCDC36→
←USP4
←AMT
11 genes omitted
48.8
49
49.2
49.4
Position on chr3 (Mb)

Supplementary Figure 2. Manhattan and quantile-quantile plots from PSC-specific genome-wide associations. The solid lines in red and the dotted lines in blue indicate the genome-wide significant two-sided unadjusted P-value of $-\log_{10}(5 \times 10^{-8})$ and the suggestive significant two-sided unadjusted P-value of $-\log_{10}(1 \times 10^{-5})$, respectively. **(a, e)** the single-trait GWAS (**GWAS_PSC**); **(b, f)** MTAG-identified PSC GWAS against five immune-mediated disorders, CD, UC, IBD, lupus, and PBC (**MTAG_PSC**); **(c, g)** MTAG PSC GWAS excluding IBD (**MTAG_PSC⊥IBD**); **(d, h)** MTAG PSC GWAS in the replicate phase (**MTAG_PSC_R**).

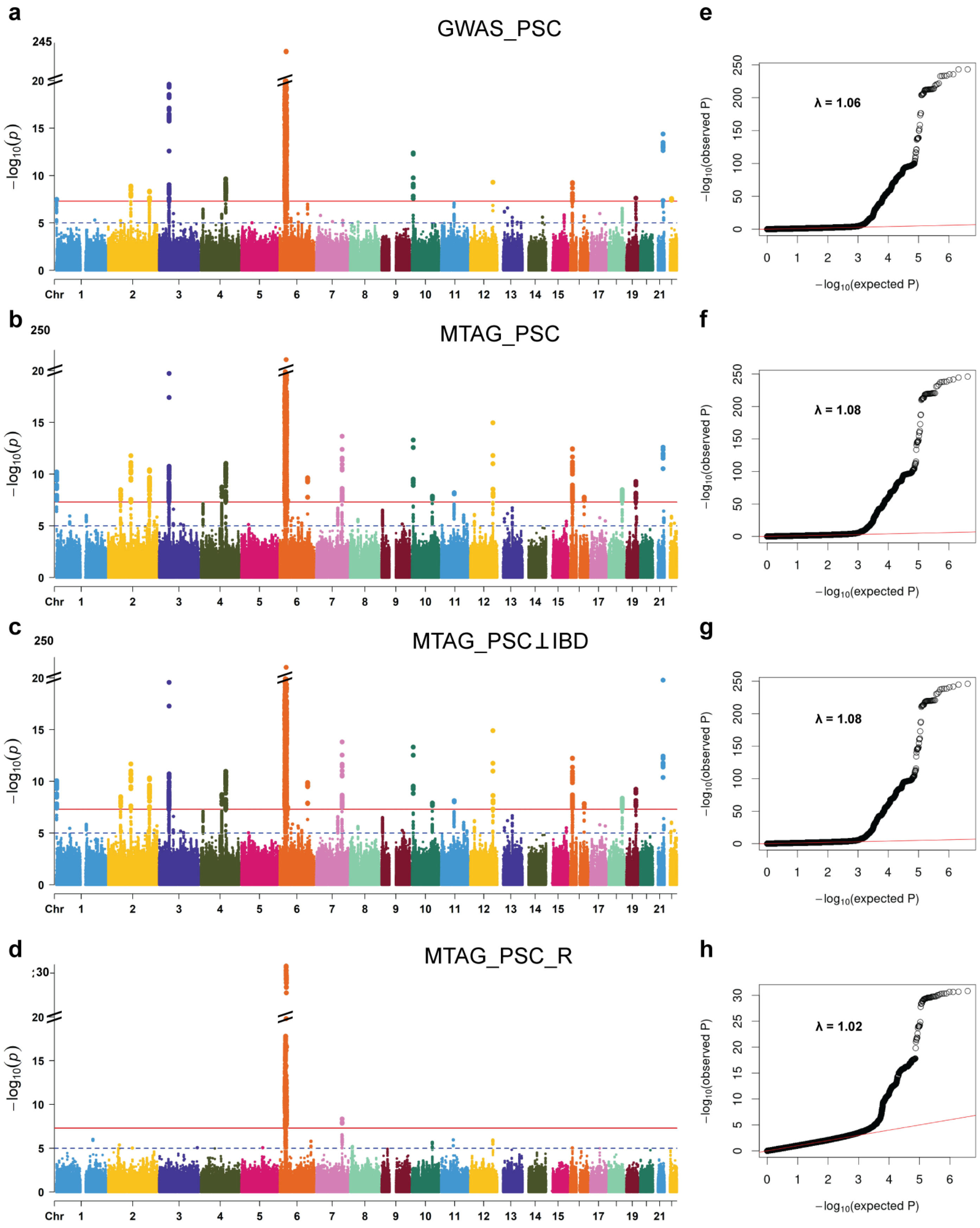
a
GWAS_PSC
b
MTAG_PSC
c
MTAG_PSC⊥IBD
d
MTAG_PSC_R
e
λ = 1.06
f
λ = 1.08
g
λ = 1.08
h
λ = 1.02
−log10(p)
Chr
−log10(observed P)
−log10(expected P)

Supplementary Figure 3. Interactions of chromatin and eQTL within PSC risk loci. Genes mapped by either Hi-C (orange), or eQTL (green) are shown on the inner circle. Genes mapped by both Hi-C and eQTL are shown in red on the inner circle. The most outer layer represents Manhattan plot including only SNPs with $P < 0.05$. SNPs in genomic risk loci are color-coded as a function of their maximum LD r^2 to the one of the independent significant SNPs in each locus, as follows: $r^2 > 0.8$ in red, $r^2 > 0.6$ in orange, $r^2 > 0.4$ in green, $r^2 > 0.2$ in blue. SNPs not in LD with any of the independent significant SNPs ($r^2 \leq 0.2$) are grey. The rsIDs of the top SNPs in each risk locus are displayed in the most outer layer. Y-axis ranges from 0 to the maximum -log10(P-value) of the SNPs. The second layer displays the chromosome ring and genomic risk locus highlighted in blue. The third layer aligns the position of genes with genomic coordinate and genomic risk locus in blue. (**a**) 2p16.1; (**b**) 3p21.31; (**c**) 4q24; (**d**) 6p21.2; (**e**) 7q32.1; (**f**) 10q24.2, (**g**) 16q22.1; (**f**) 21q22.2.

a

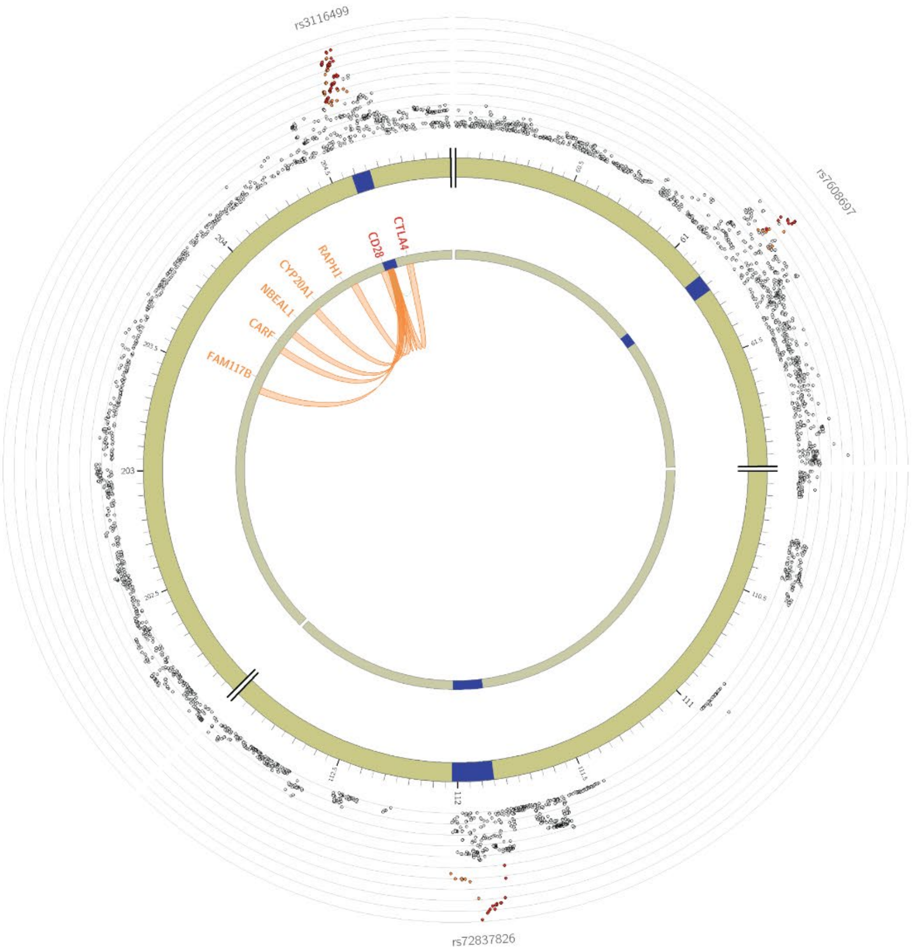
rs3116499
rs7608697
rs72837826
CTLA4
CD28
RAPH1
CYP20A1
NBEAL1
CARF
FAM117B
204
203.5
203
202.5
61
61.5
110.5
111
111.5
112
112.5

b

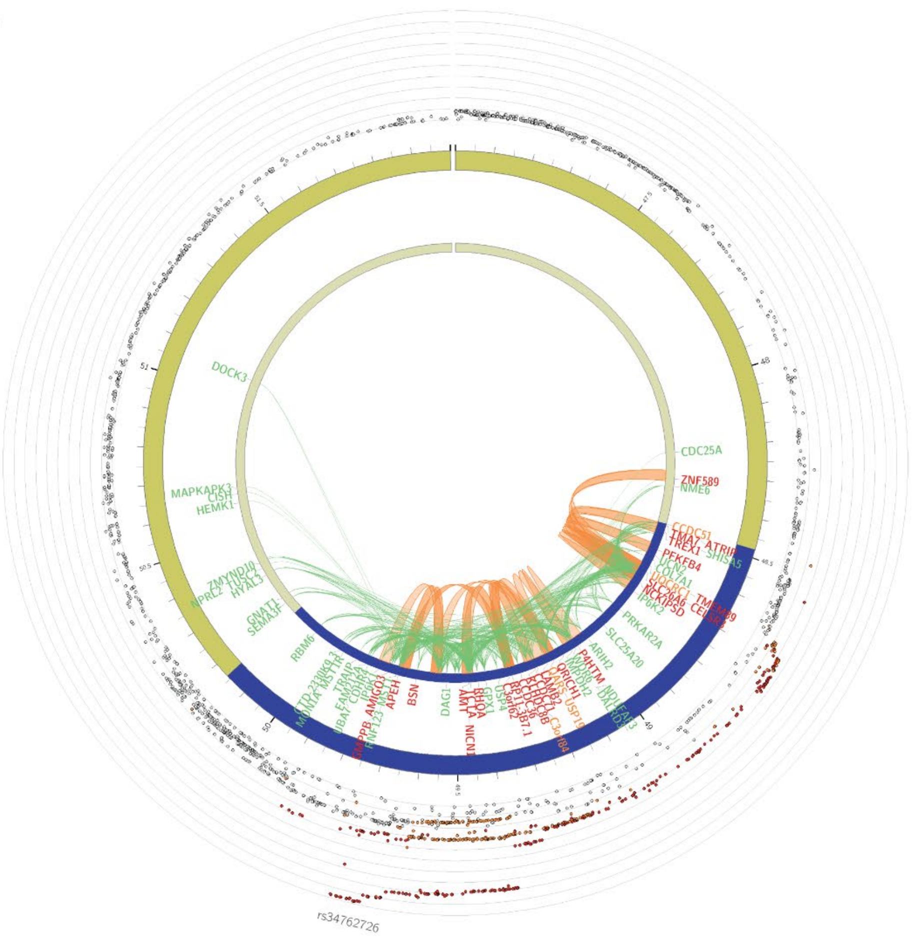
DOCK3
CDC25A
ZNF589
NME6
MAPKAPK3
CISH
HEMK1
CCDC51
TREX1
PFKFB4
ZMYND10
HYAL3
GNAT1
SEMA3F
RBM6
PRKAR2A
SLC25A20
ARIH2
P4HTM
BSN
DAG1
APEH
rs34762726
51
50.5
50
48
48.5
49

c

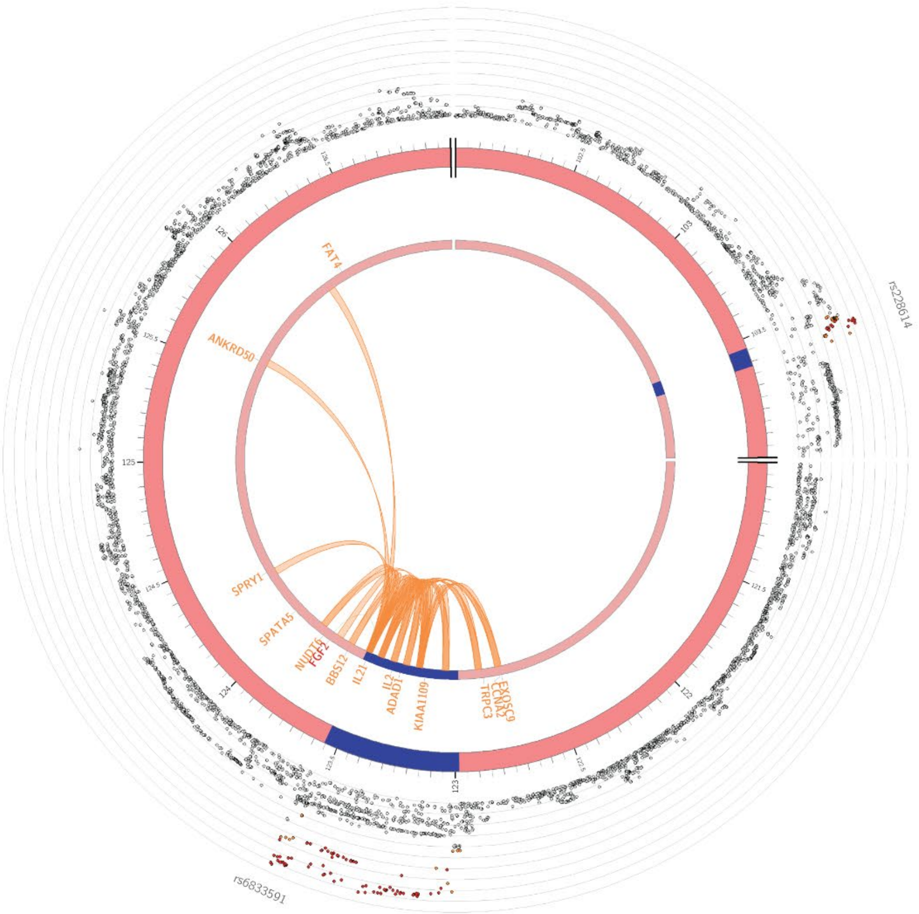
rs228614
rs6833591
FAT4
ANKRD50
SPRY1
SPATA5
NUDT6
FGF2
BBS12
IL21
IL2
ADAD1
KIAA1109
TRPC3
CCNA2
EXOSC9

d

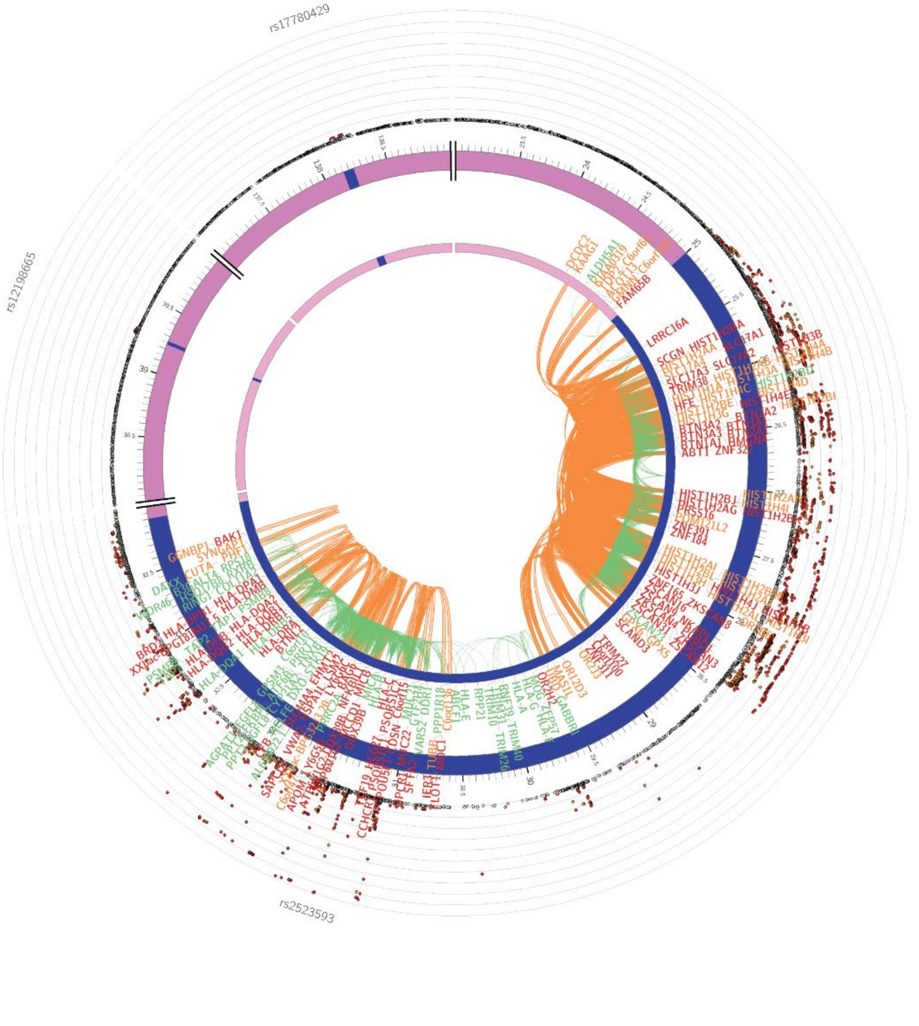
rs17780429
rs12198665
rs2523593
LRRC16A
ZNF391
ZNF184
PRSS16

e

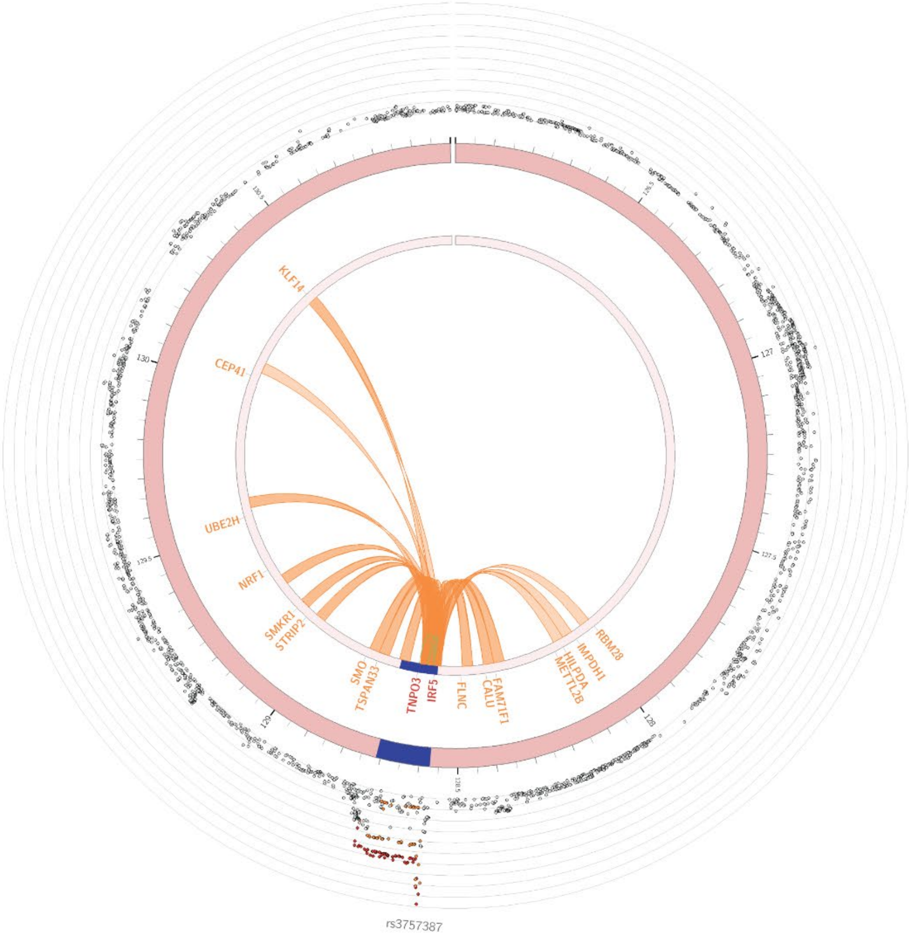
KLF14
CEP41
UBE2H
NRF1
SMKR1
STRIP2
SMO
TSPAN33
TNPO3
IRF5
FLNC
CALU
FAM71F1
METTL2B
HILPDA
IMPDH1
RBM28
rs3757387

f

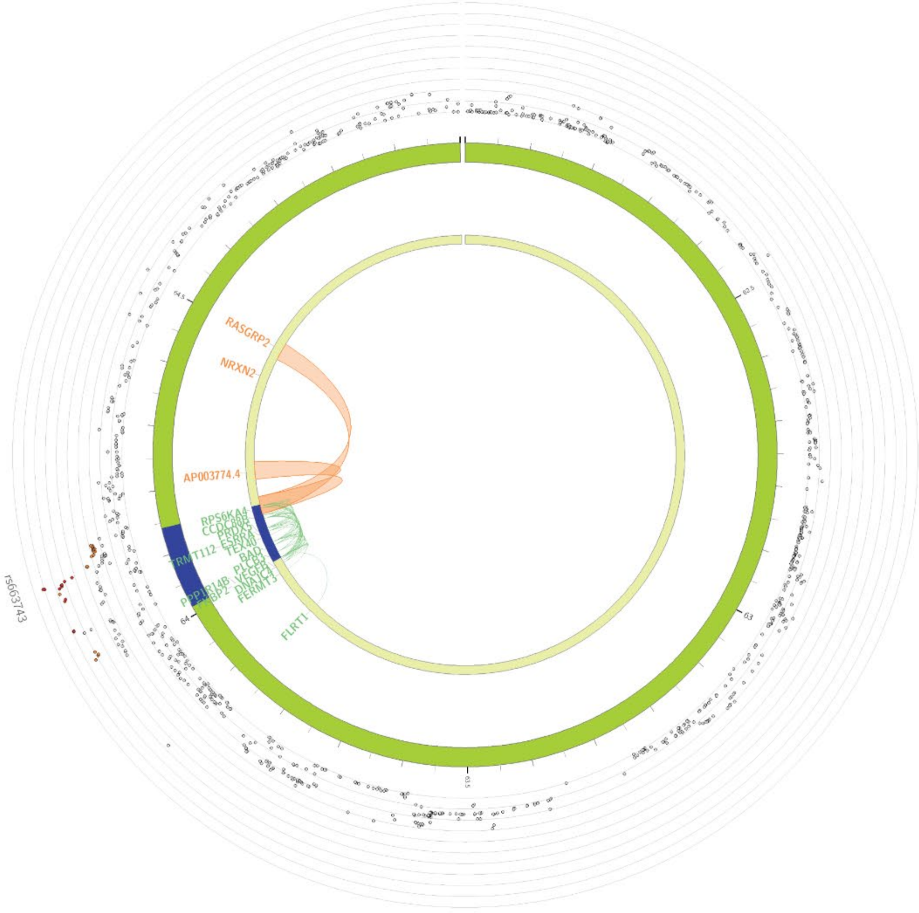
RASGRP2
NRXN2
AP003774.4
RPS6KA4
TRMT112
PPP1R14B
PLCB3
VEGFB
DNAJC4
FERMT3
FLRT1
rs663743

g

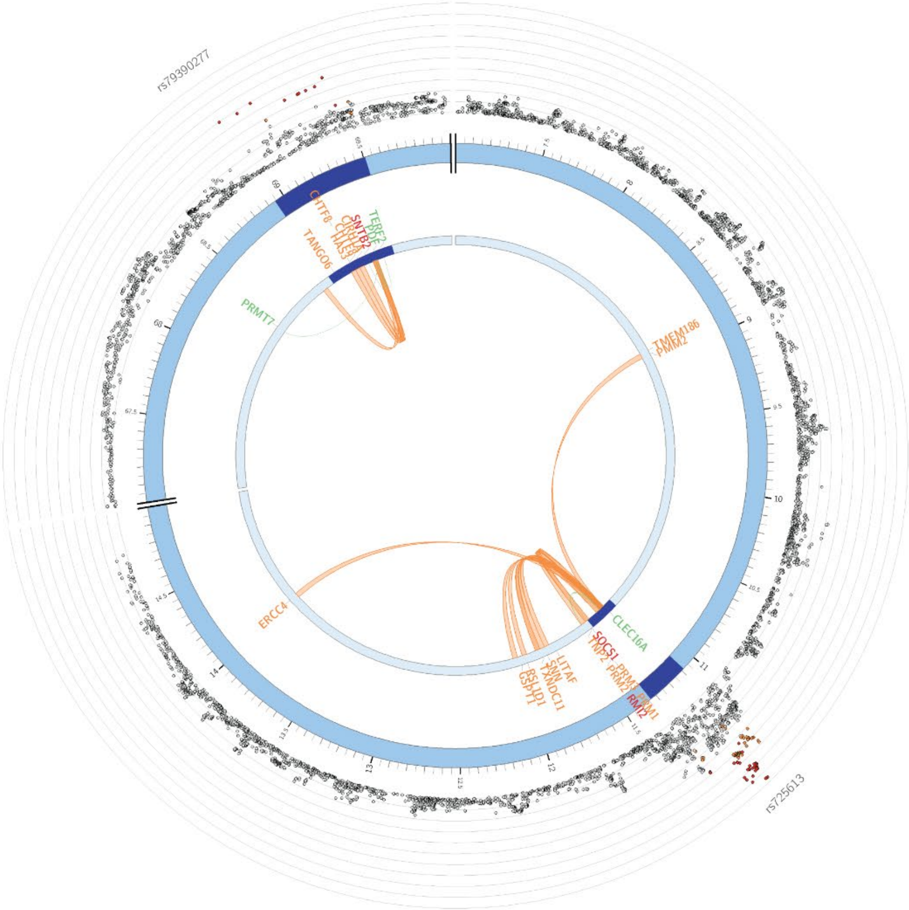
rs79390277
rs725613
TEBP2
SNTB2
TANGO6
PRMT7
TMEM186
PMM2
ERCC4
CLEC16A
SOCS1
LITAF
ETS2
KCNJ15
DYRK1A

h

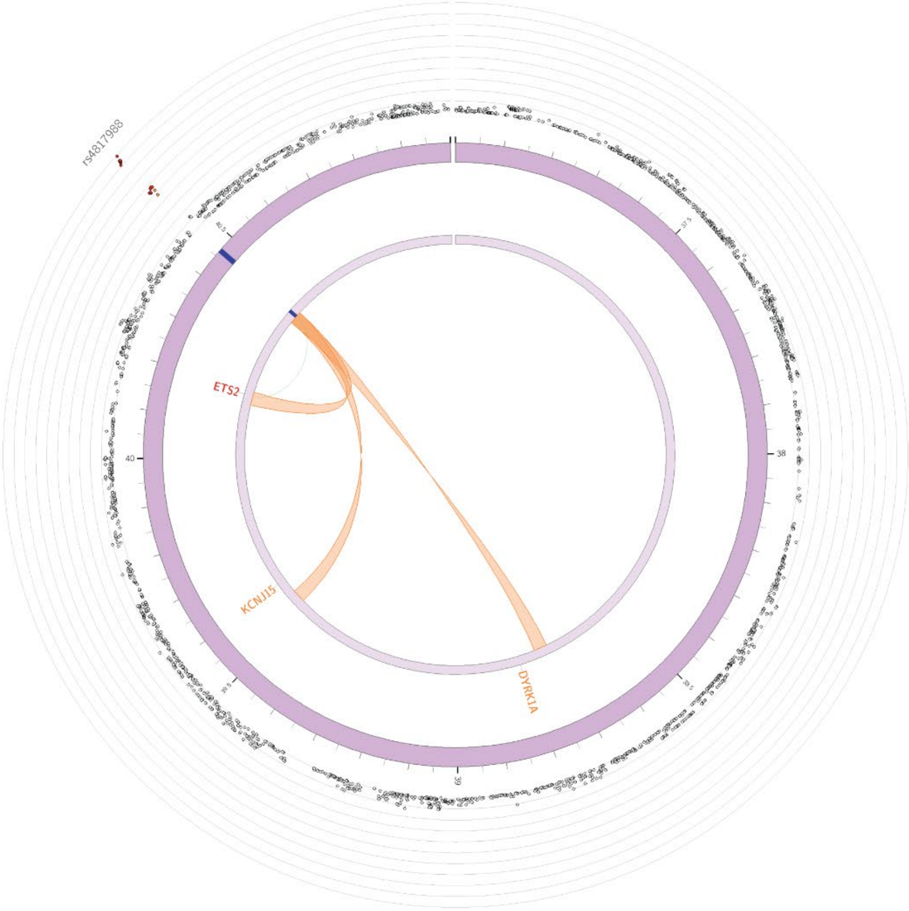
rs4817988
ETS2
KCNJ15
DYRK1A

Supplementary Figure 4. **Colocalization plots between PSC MTAG and eQTL tissue**. Functional validation of the MTAG-identified PSC candidate genes suggests the plausible posterior probability that both PSC and tested tissue are associated and share a single function variant (PP4) at the threshold of PP4 > 0.80. (**a1-a3**) Tissue-specific colocalization for ***MANBA***; (b**C1-b30**) for ***IRF5***; (**c1-c3**) for ***NKX2-3***.

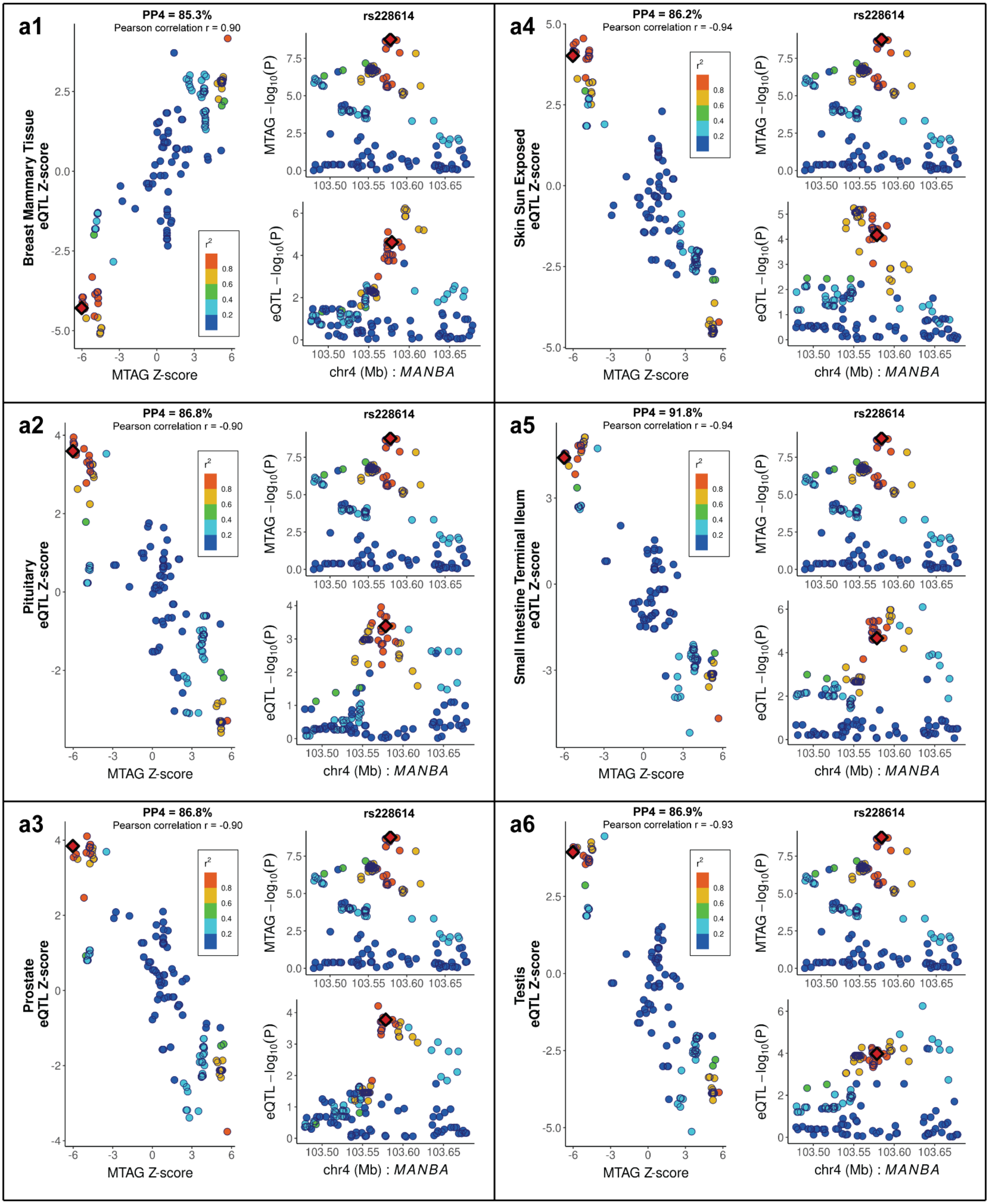

a1
PP4 = 85.3%
Pearson correlation r = 0.90
Breast Mammary Tissue eQTL Z-score
MTAG Z-score
rs228614
MTAG −log10(P)
eQTL −log10(P)
chr4 (Mb) : MANBA
a2
PP4 = 86.8%
Pearson correlation r = -0.90
Pituitary eQTL Z-score
a3
PP4 = 86.8%
Pearson correlation r = -0.90
Prostate eQTL Z-score
a4
PP4 = 86.2%
Pearson correlation r = -0.94
Skin Sun Exposed eQTL Z-score
a5
PP4 = 91.8%
Pearson correlation r = -0.94
Small Intestine Terminal Ileum eQTL Z-score
a6
PP4 = 86.9%
Pearson correlation r = -0.93
Testis eQTL Z-score
r2
0.8
0.6
0.4
0.2

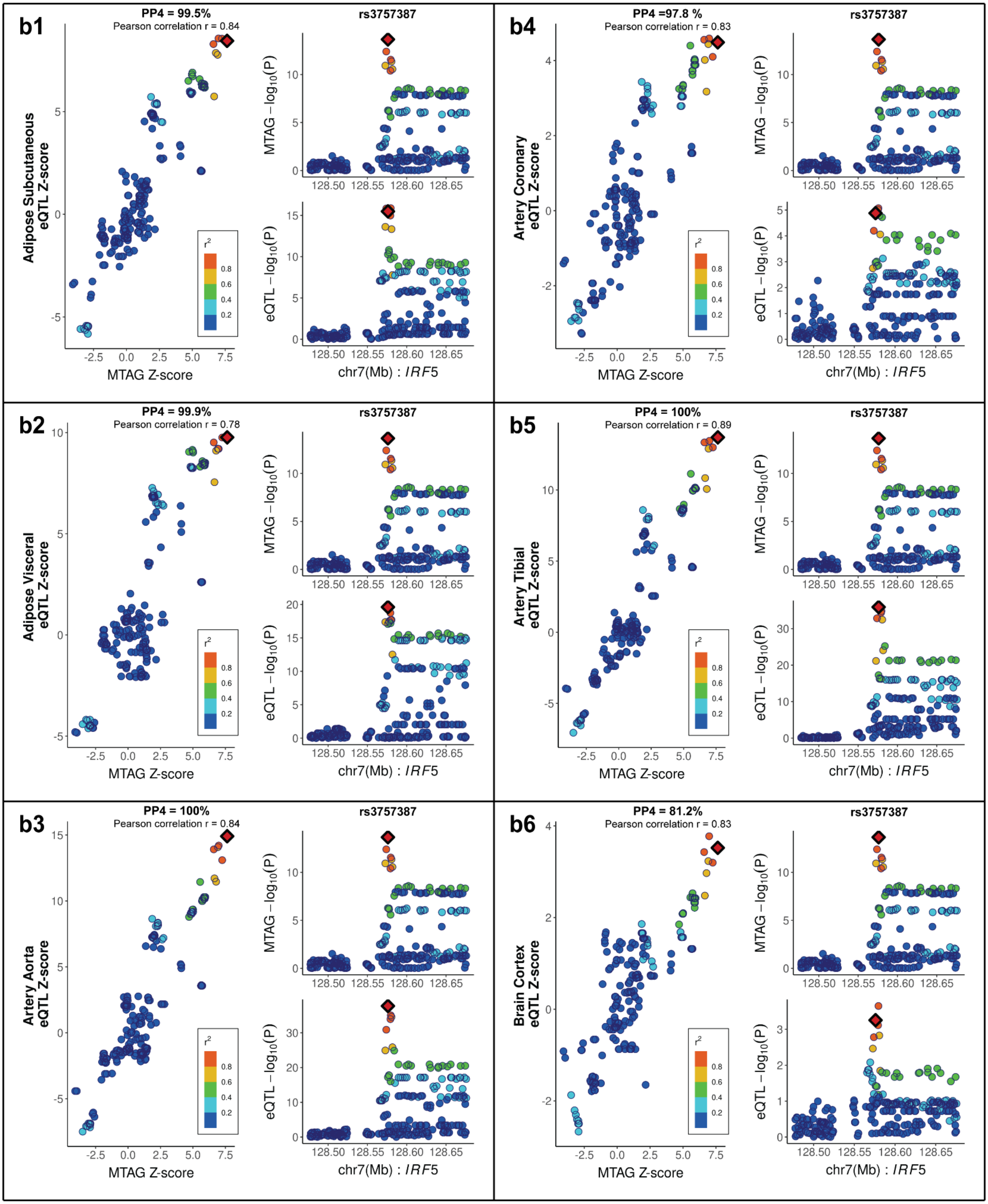

b1
PP4 = 99.5%
Pearson correlation r = 0.84
rs3757387
Adipose Subcutaneous eQTL Z-score
MTAG Z-score
MTAG −log10(P)
eQTL −log10(P)
chr7(Mb) : IRF5
b2
PP4 = 99.9%
Pearson correlation r = 0.78
Adipose Visceral eQTL Z-score
b3
PP4 = 100%
Pearson correlation r = 0.84
Artery Aorta eQTL Z-score
b4
PP4 =97.8 %
Pearson correlation r = 0.83
Artery Coronary eQTL Z-score
b5
PP4 = 100%
Pearson correlation r = 0.89
Artery Tibial eQTL Z-score
b6
PP4 = 81.2%
Pearson correlation r = 0.83
Brain Cortex eQTL Z-score

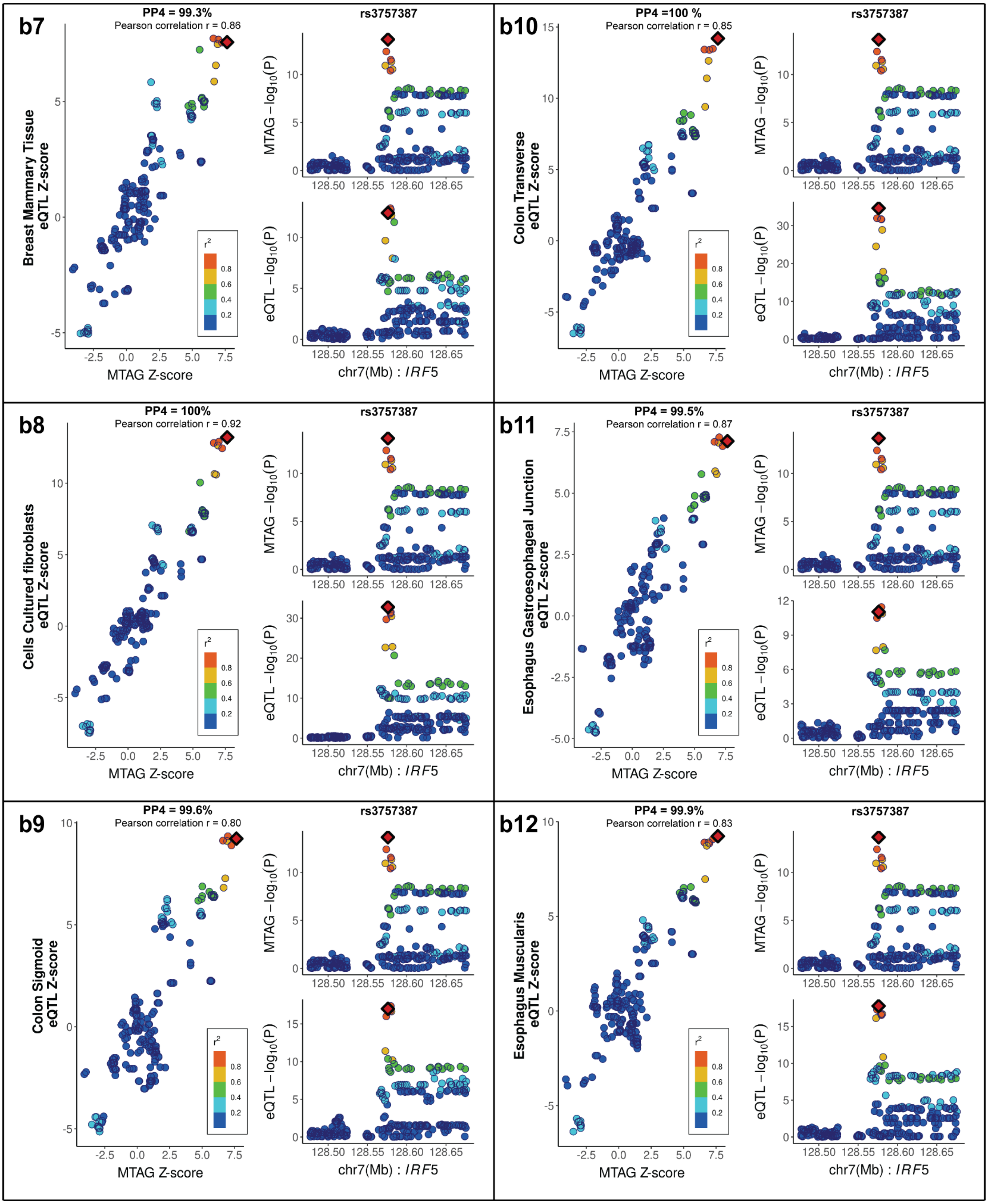
b7
PP4 = 99.3%
Pearson correlation r = 0.86
Breast Mammary Tissue eQTL Z-score
MTAG Z-score
rs3757387
MTAG −log10(P)
eQTL −log10(P)
chr7(Mb) : IRF5
b10
PP4 =100 %
Pearson correlation r = 0.85
Colon Transverse eQTL Z-score
MTAG Z-score
rs3757387
MTAG −log10(P)
eQTL −log10(P)
chr7(Mb) : IRF5
b8
PP4 = 100%
Pearson correlation r = 0.92
Cells Cultured fibroblasts eQTL Z-score
MTAG Z-score
rs3757387
MTAG −log10(P)
eQTL −log10(P)
chr7(Mb) : IRF5
b11
PP4 = 99.5%
Pearson correlation r = 0.87
Esophagus Gastroesophageal Junction eQTL Z-score
MTAG Z-score
rs3757387
MTAG −log10(P)
eQTL −log10(P)
chr7(Mb) : IRF5
b9
PP4 = 99.6%
Pearson correlation r = 0.80
Colon Sigmoid eQTL Z-score
MTAG Z-score
rs3757387
MTAG −log10(P)
eQTL −log10(P)
chr7(Mb) : IRF5
b12
PP4 = 99.9%
Pearson correlation r = 0.83
Esophagus Muscularis eQTL Z-score
MTAG Z-score
rs3757387
MTAG −log10(P)
eQTL −log10(P)
chr7(Mb) : IRF5
r2
0.8
0.6
0.4
0.2

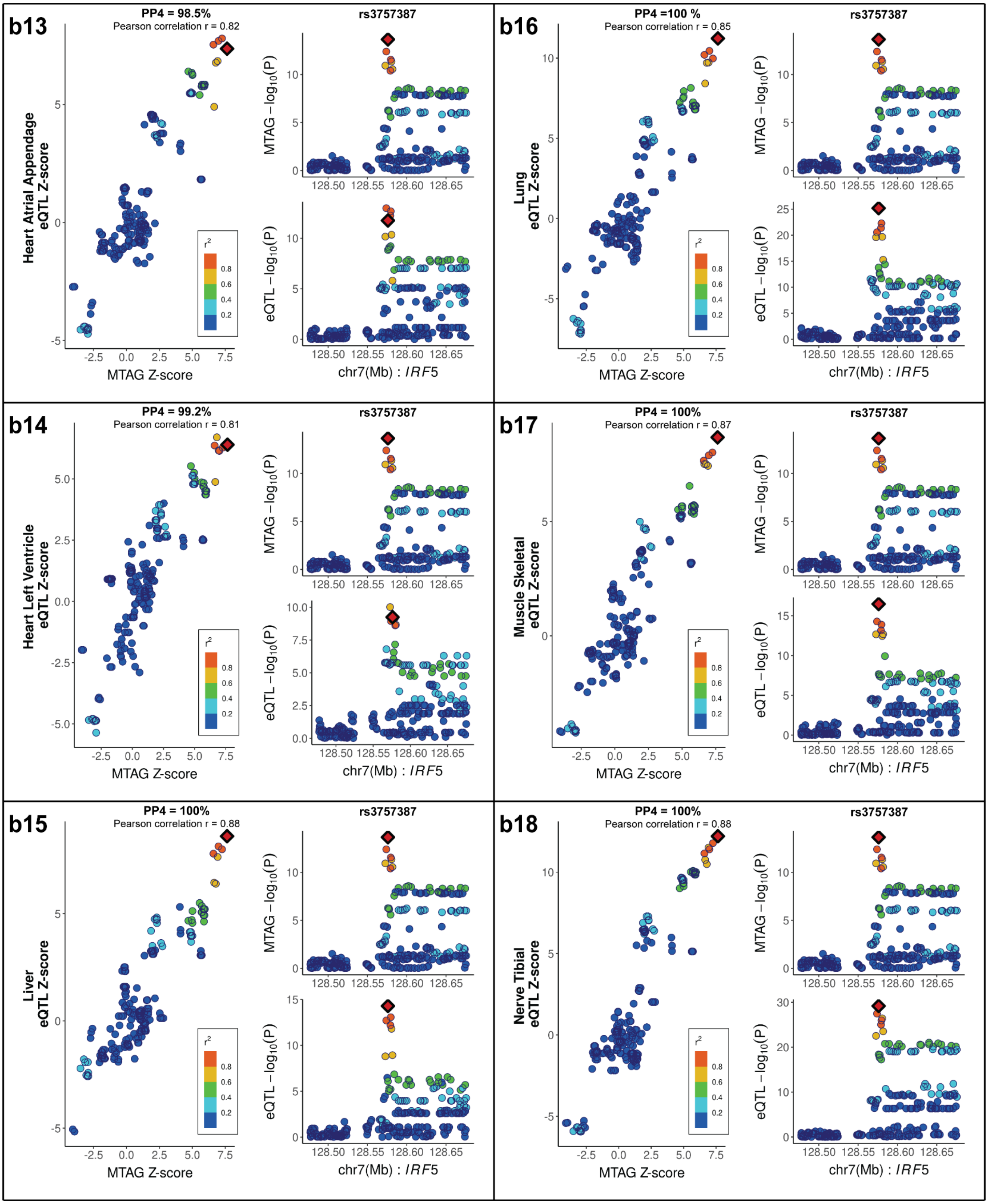
b13
PP4 = 98.5%
Pearson correlation r = 0.82
rs3757387
Heart Atrial Appendage eQTL Z-score
MTAG Z-score
MTAG −log10(P)
eQTL −log10(P)
chr7(Mb) : IRF5
r2
b16
PP4 =100 %
Pearson correlation r = 0.85
rs3757387
Lung eQTL Z-score
MTAG Z-score
MTAG −log10(P)
eQTL −log10(P)
chr7(Mb) : IRF5
b14
PP4 = 99.2%
Pearson correlation r = 0.81
rs3757387
Heart Left Ventricle eQTL Z-score
MTAG Z-score
MTAG −log10(P)
eQTL −log10(P)
chr7(Mb) : IRF5
b17
PP4 = 100%
Pearson correlation r = 0.87
rs3757387
Muscle Skeletal eQTL Z-score
MTAG Z-score
MTAG −log10(P)
eQTL −log10(P)
chr7(Mb) : IRF5
b15
PP4 = 100%
Pearson correlation r = 0.88
rs3757387
Liver eQTL Z-score
MTAG Z-score
MTAG −log10(P)
eQTL −log10(P)
chr7(Mb) : IRF5
b18
PP4 = 100%
Pearson correlation r = 0.88
rs3757387
Nerve Tibial eQTL Z-score
MTAG Z-score
MTAG −log10(P)
eQTL −log10(P)
chr7(Mb) : IRF5

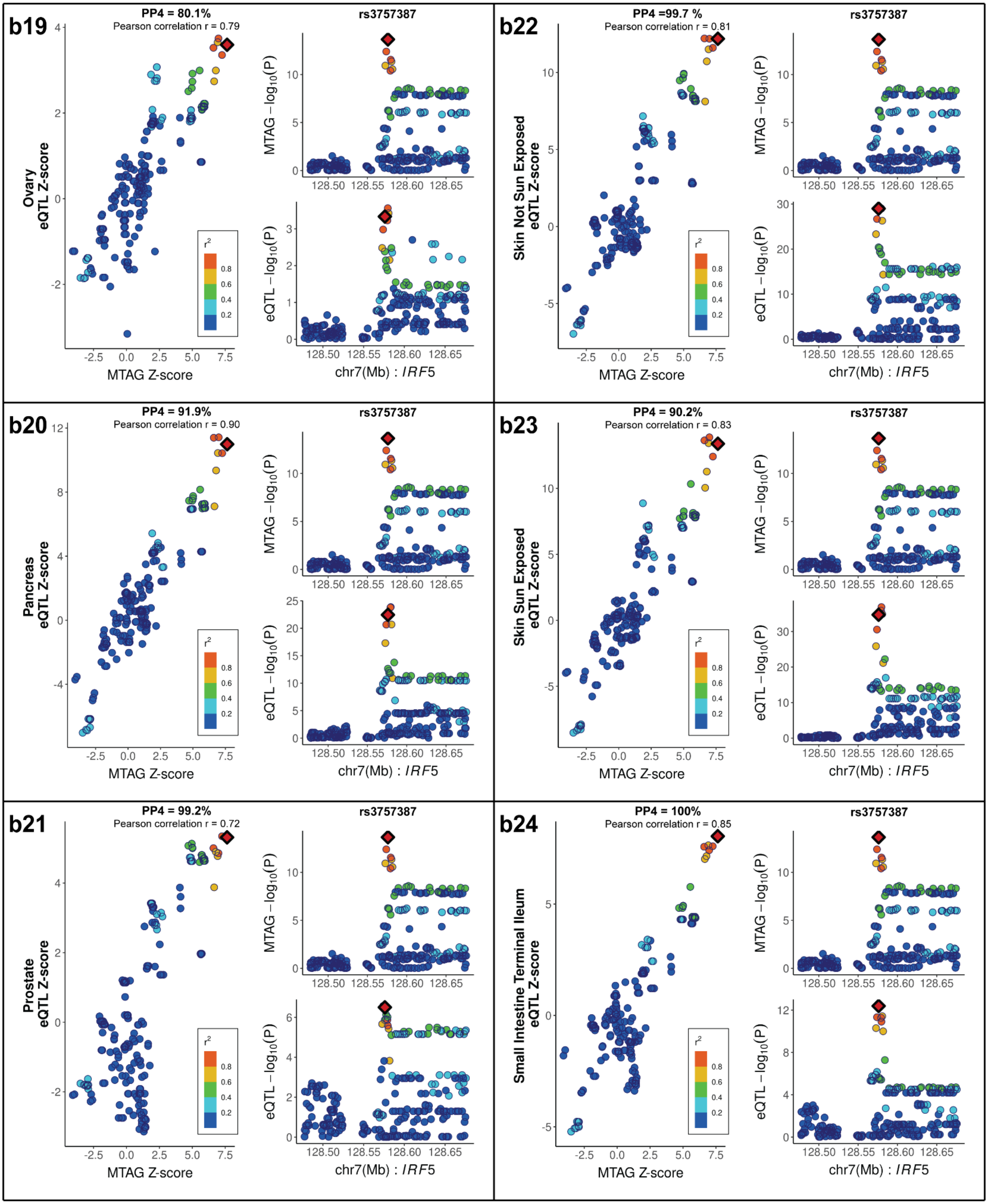
b19
PP4 = 80.1%
Pearson correlation r = 0.79
Ovary eQTL Z-score
MTAG Z-score
rs3757387
MTAG −log10(P)
eQTL −log10(P)
chr7(Mb) : IRF5
b22
PP4 =99.7 %
Pearson correlation r = 0.81
Skin Not Sun Exposed eQTL Z-score
MTAG Z-score
rs3757387
MTAG −log10(P)
eQTL −log10(P)
chr7(Mb) : IRF5
b20
PP4 = 91.9%
Pearson correlation r = 0.90
Pancreas eQTL Z-score
MTAG Z-score
rs3757387
MTAG −log10(P)
eQTL −log10(P)
chr7(Mb) : IRF5
b23
PP4 = 90.2%
Pearson correlation r = 0.83
Skin Sun Exposed eQTL Z-score
MTAG Z-score
rs3757387
MTAG −log10(P)
eQTL −log10(P)
chr7(Mb) : IRF5
b21
PP4 = 99.2%
Pearson correlation r = 0.72
Prostate eQTL Z-score
MTAG Z-score
rs3757387
MTAG −log10(P)
eQTL −log10(P)
chr7(Mb) : IRF5
b24
PP4 = 100%
Pearson correlation r = 0.85
Small Intestine Terminal Ileum eQTL Z-score
MTAG Z-score
rs3757387
MTAG −log10(P)
eQTL −log10(P)
chr7(Mb) : IRF5
r2
0.8
0.6
0.4
0.2

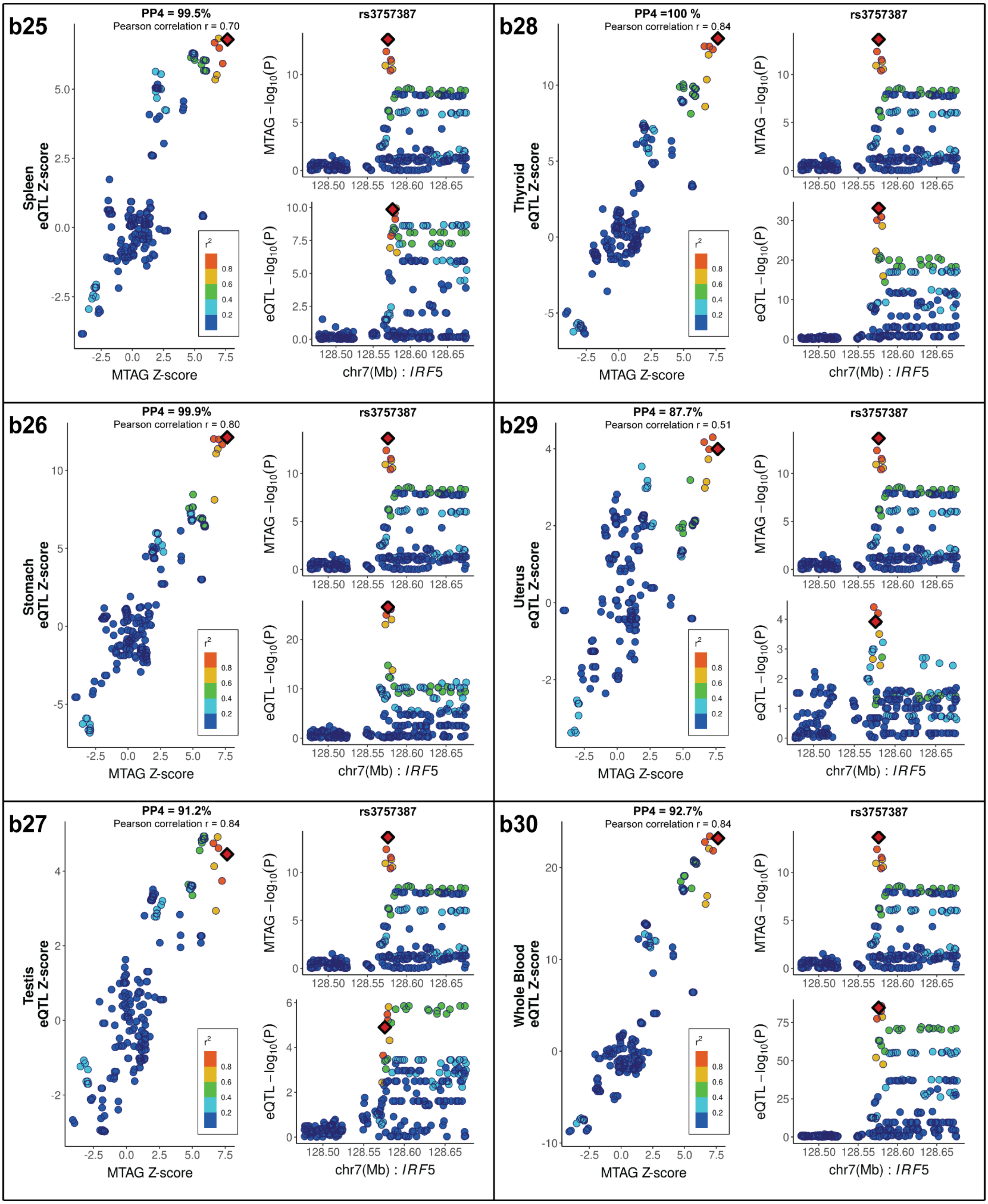
b25
PP4 = 99.5%
Pearson correlation r = 0.70
rs3757387
Spleen eQTL Z-score
MTAG Z-score
MTAG −log10(P)
eQTL −log10(P)
chr7(Mb) : IRF5
b28
PP4 =100 %
Pearson correlation r = 0.84
rs3757387
Thyroid eQTL Z-score
MTAG Z-score
MTAG −log10(P)
eQTL −log10(P)
chr7(Mb) : IRF5
b26
PP4 = 99.9%
Pearson correlation r = 0.80
rs3757387
Stomach eQTL Z-score
MTAG Z-score
MTAG −log10(P)
eQTL −log10(P)
chr7(Mb) : IRF5
b29
PP4 = 87.7%
Pearson correlation r = 0.51
rs3757387
Uterus eQTL Z-score
MTAG Z-score
MTAG −log10(P)
eQTL −log10(P)
chr7(Mb) : IRF5
b27
PP4 = 91.2%
Pearson correlation r = 0.84
rs3757387
Testis eQTL Z-score
MTAG Z-score
MTAG −log10(P)
eQTL −log10(P)
chr7(Mb) : IRF5
b30
PP4 = 92.7%
Pearson correlation r = 0.84
rs3757387
Whole Blood eQTL Z-score
MTAG Z-score
MTAG −log10(P)
eQTL −log10(P)
chr7(Mb) : IRF5
r2
0.8
0.6
0.4
0.2

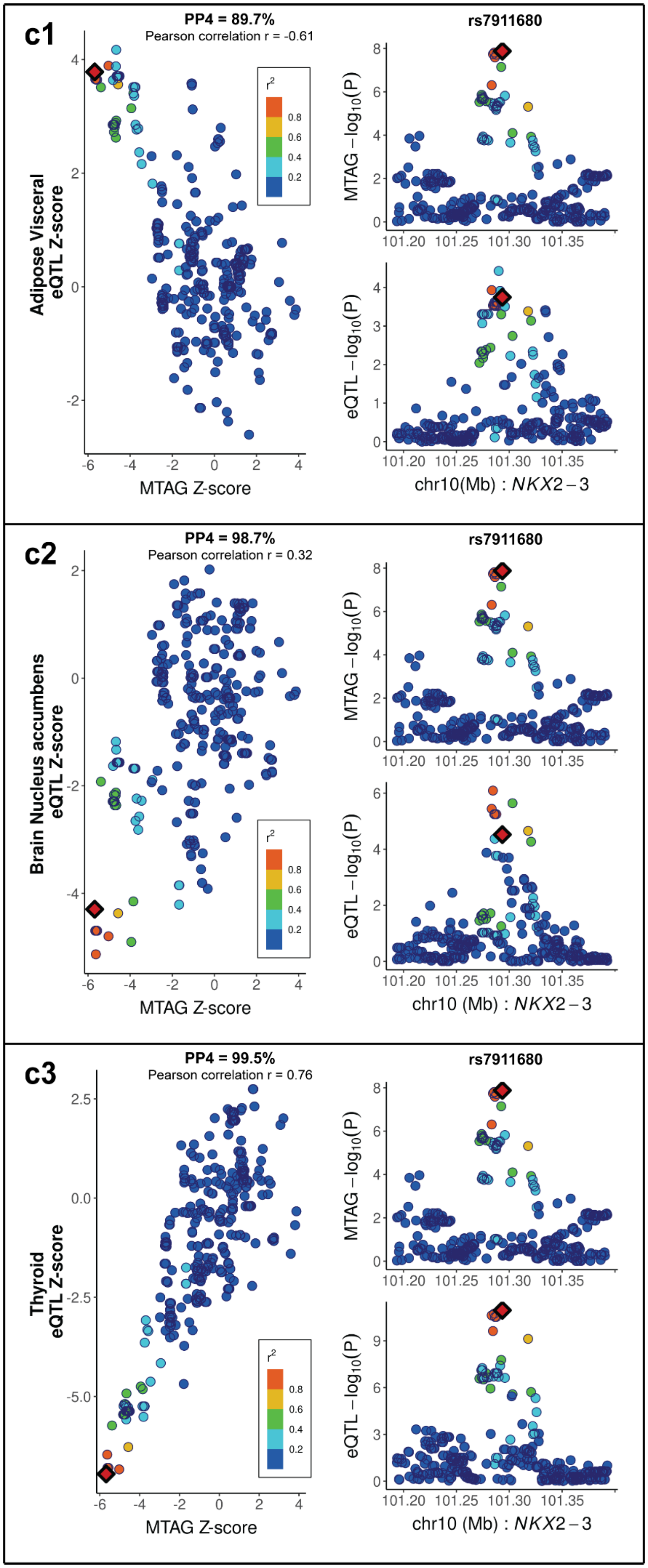
c1
PP4 = 89.7%
Pearson correlation r = -0.61
rs7911680
Adipose Visceral eQTL Z-score
MTAG Z-score
MTAG −log10(P)
eQTL −log10(P)
chr10(Mb) : NKX2−3
c2
PP4 = 98.7%
Pearson correlation r = 0.32
rs7911680
Brain Nucleus accumbens eQTL Z-score
MTAG Z-score
MTAG −log10(P)
eQTL −log10(P)
chr10 (Mb) : NKX2−3
c3
PP4 = 99.5%
Pearson correlation r = 0.76
rs7911680
Thyroid eQTL Z-score
MTAG Z-score
MTAG −log10(P)
eQTL −log10(P)
chr10 (Mb) : NKX2−3

Supplementary Figure 5. **STRING protein-protein interaction (PPI) plots modeled in STRING using 406 candidate genes.** The prioritized 406 genes displayed relevant groups of related genes involved in the regulation of specific biological pathways using PPI networks. Results of highly enriched pathways using STRING PPI are shown in Supplementary Data 13.

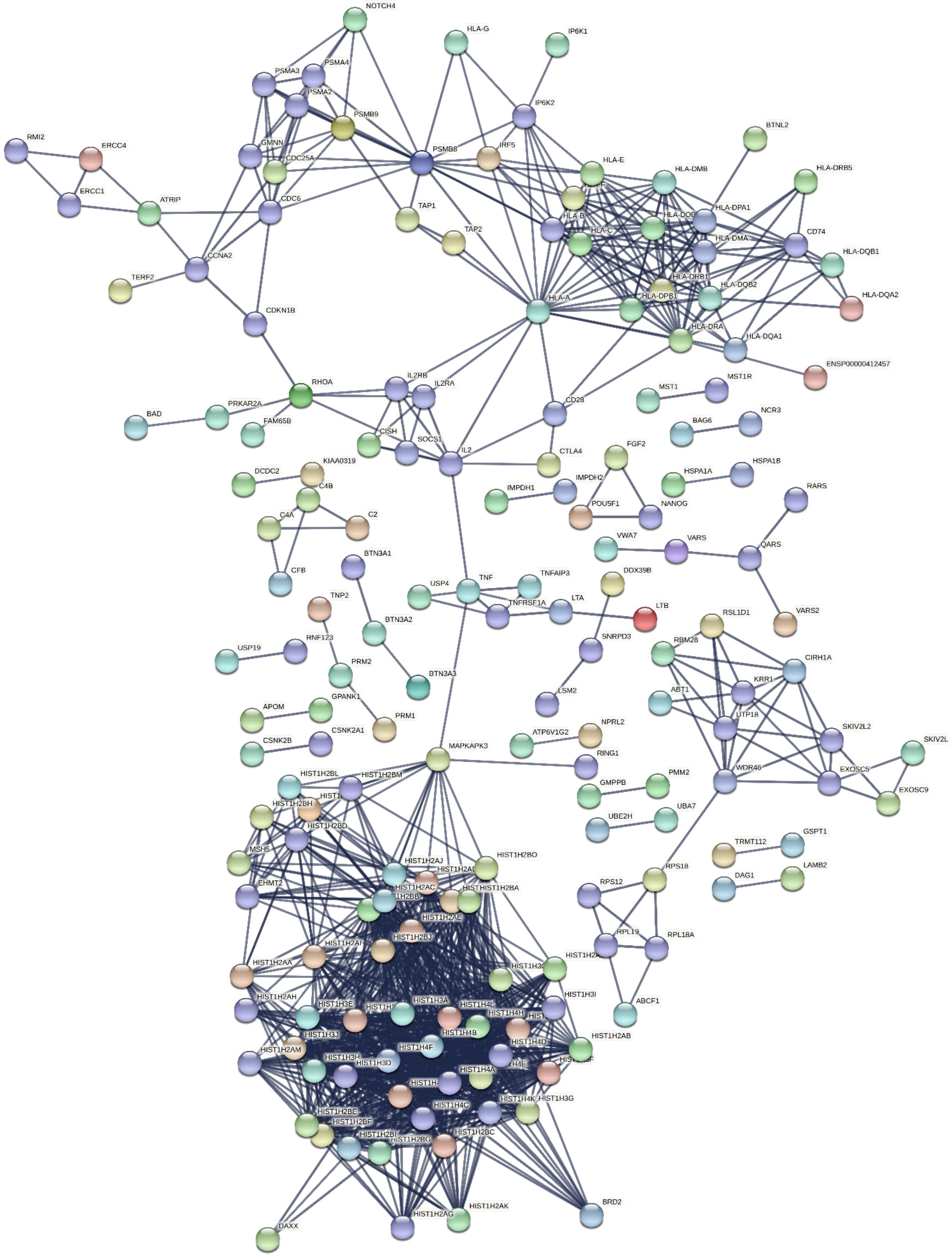
NOTCH4
HLA-G
IP6K1
PSMA3
PSMA4
PSMA2
PSMB9
IP6K2
BTNL2
RMI2
ERCC4
GMNN
PSMB8
IRF5
HLA-E
HLA-DMB
HLA-DRB5
CDC25A
ERCC1
ATRIP
CDC6
TAP1
HLA-B
HLA-DPA1
HLA-C
TAP2
HLA-DMA
CD74
HLA-DQB1
CCNA2
TERF2
HLA-DRB1
HLA-DQB2
HLA-DQA2
HLA-A
HLA-DPB1
CDKN1B
HLA-DRA
HLA-DQA1
ENSP00000412457
IL2RB
IL2RA
RHOA
MST1
MST1R
PRKAR2A
BAD
CD28
FAM65B
NCR3
BAG6
CISH
SOCS1
IL2
CTLA4
FGF2
KIAA0319
DCDC2
HSPA1A
HSPA1B
IMPDH2
C4B
IMPDH1
POU5F1
NANOG
RARS
C4A
C2
VWA7
VARS
QARS
CFB
BTN3A1
TNFAIP3
DDX39B
TNF
USP4
TNP2
TNFRSF1A
LTA
LTB
VARS2
RSL1D1
BTN3A2
RNF123
SNRPD3
RBM28
USP19
CIRH1A
PRM2
BTN3A3
KRR1
ABT1
LSM2
GPANK1
APOM
UTP18
PRM1
NPRL2
SKIV2L2
CSNK2B
CSNK2A1
ATP6V1G2
SKIV2L
MAPKAPK3
RING1
WDR46
EXOSC5
HIST1H2BL
HIST1H2BM
PMM2
GMPPB
EXOSC9
HIST1H2BH
HIST1H2BD
UBA7
UBE2H
GSPT1
TRMT112
MSH5
HIST1H2BO
HIST1H2AJ
RPS18
LAMB2
DAG1
RPS12
EHMT2
HIST1H2AC
HIST1H2BA
HIST1H2AE
HIST1H2BJ
RPL19
RPL18A
HIST1H2AI
HIST1H2AA
HIST1H3I
HIST1H2AH
HIST1H3E
HIST1H3A
HIST1H4L
HIST1H4H
ABCF1
HIST1H3J
HIST1H4B
HIST1H4D
HIST1H2AB
HIST1H2AM
HIST1H4F
HIST1H3H
HIST1H3D
HIST1H4A
HIST1H4E
HIST1H4K
HIST1H3G
HIST1H4C
HIST1H2BE
HIST1H2BF
HIST1H2BI
HIST1H2BG
HIST1H2BC
BRD2
HIST1H2AK
HIST1H2AG
DAXX

Supplementary Figure 6. **STRING protein-protein interaction (PPI) plots modeled in STRING.** MTAG-identified PSC-specific candidate gene network reported for (**a**) ***MANBA*** (PPI enrichment P-value, $P=5.16\times10^{-14}$); (**b**) for ***IRF5*** ($P=1.00\times10^{-16}$); (**c**) for ***NKX2-3*** ($P=1.13\times10^{-9}$).

a

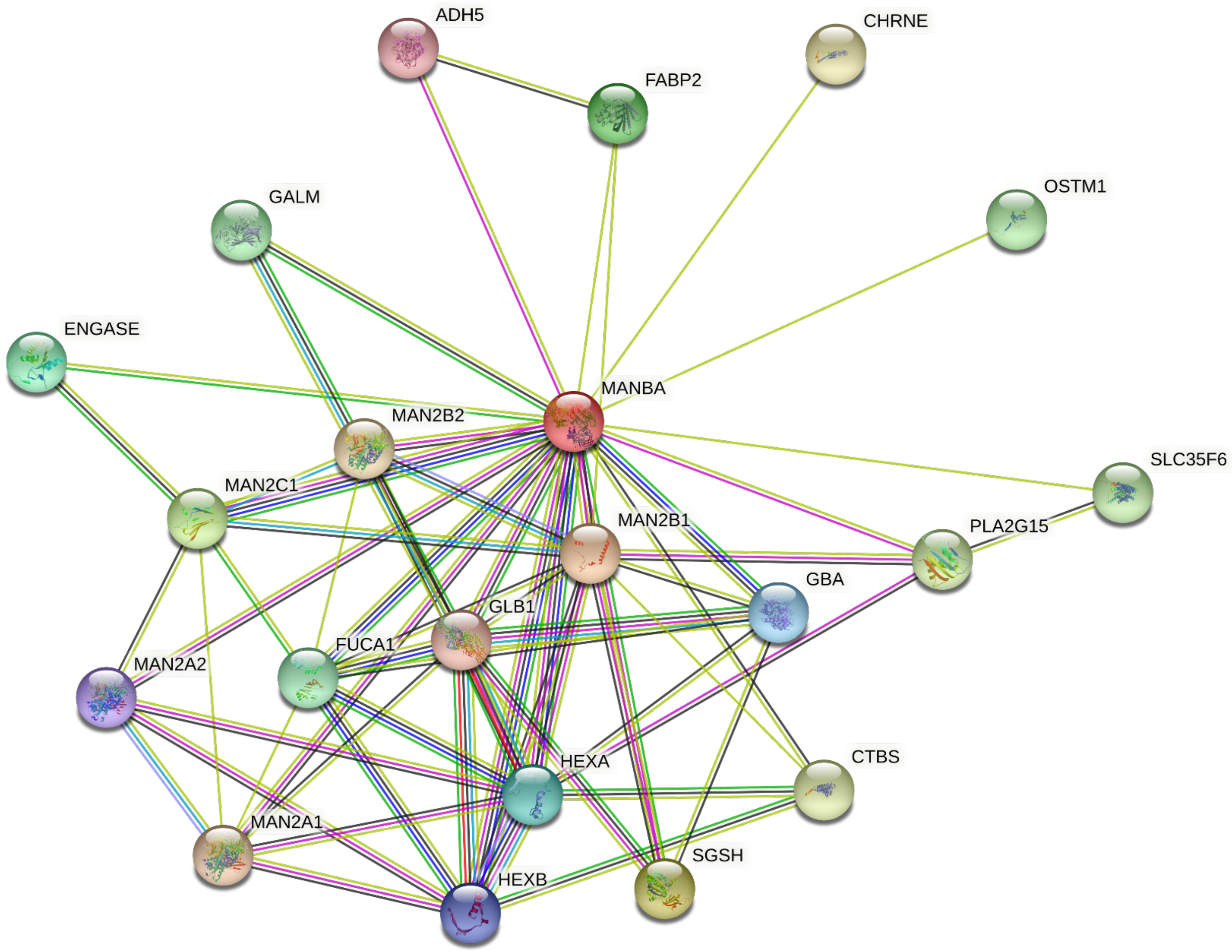

b

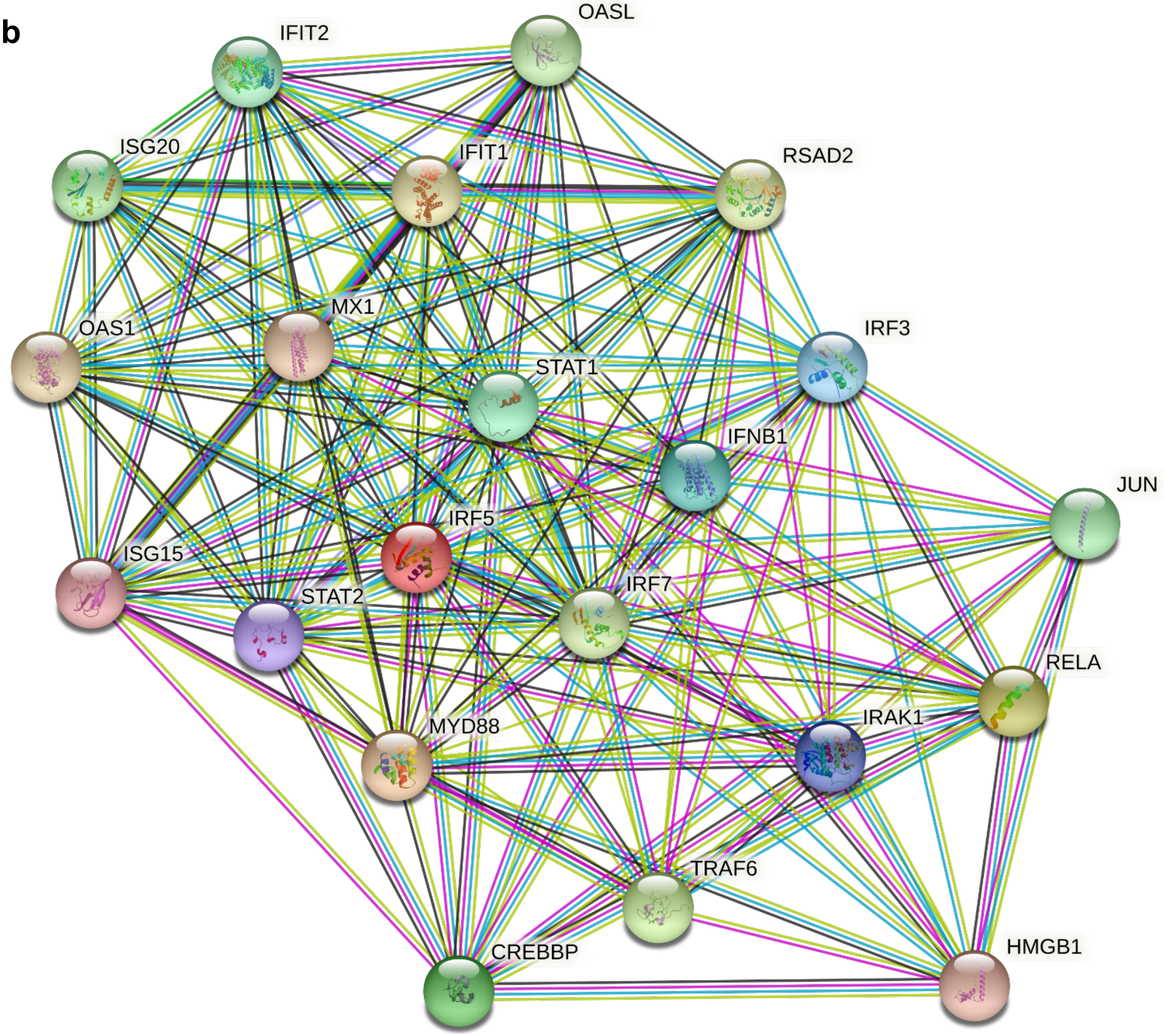

IFIT2
OASL
ISG20
IFIT1
RSAD2
MX1
OAS1
STAT1
IRF3
IFNB1
JUN
IRF5
ISG15
STAT2
IRF7
RELA
MYD88
IRAK1
TRAF6
HMGB1
CREBBP

c

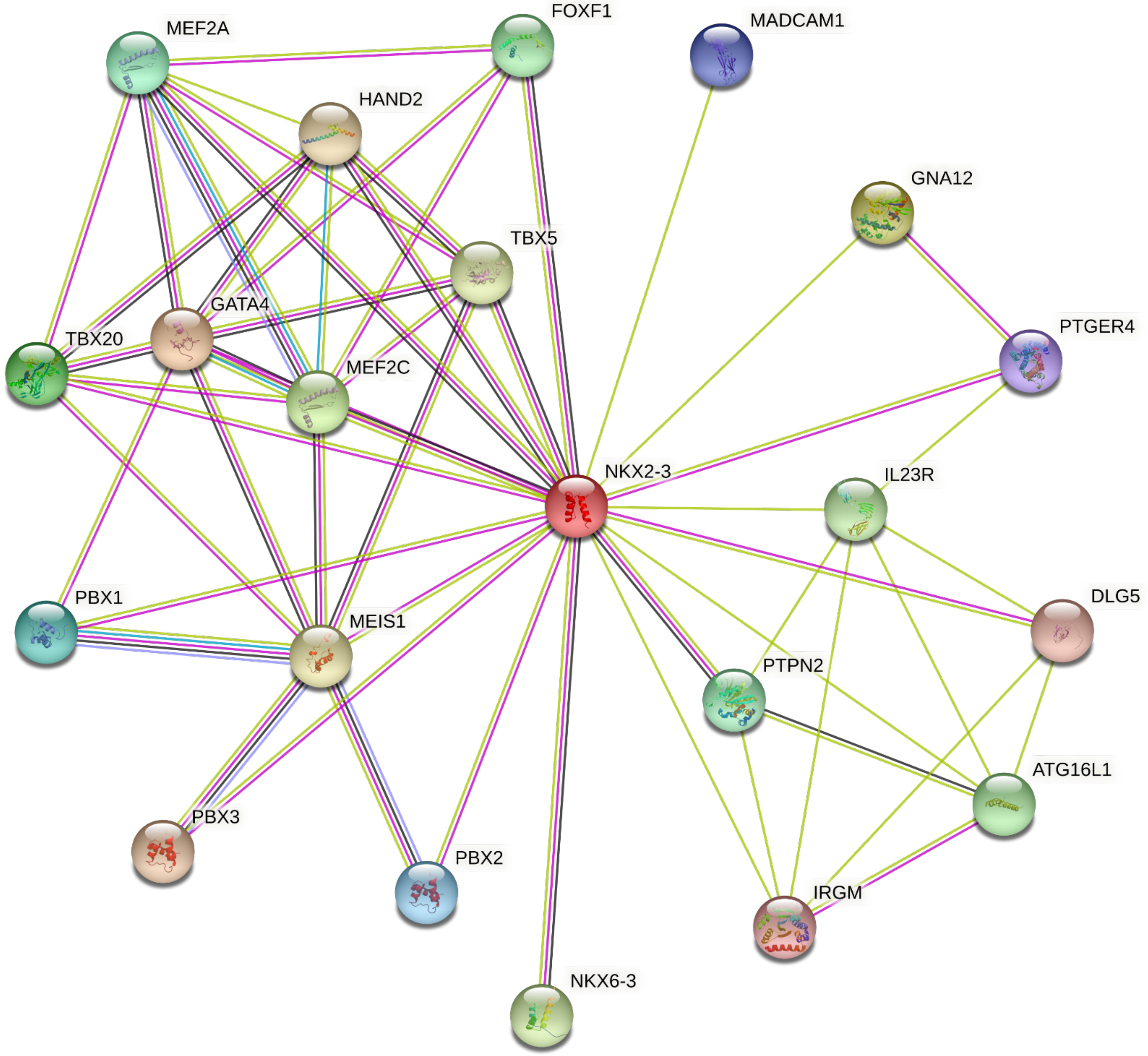

MEF2A
FOXF1
MADCAM1
HAND2
GNA12
TBX5
GATA4
TBX20
MEF2C
PTGER4
NKX2-3
IL23R
PBX1
MEIS1
DLG5
PTPN2
ATG16L1
PBX3
PBX2
IRGM
NKX6-3
