# Supplementary material for: Multitrait genome-wide analyses identify new susceptibility loci and candidate drugs to primary sclerosing cholangitis
Source: Nat Commun. 2023 Feb 24;14:1069. doi: 10.1038/s41467-023-36678-8 (PMC9958016; doi:10.1038/s41467-023-36678-8)
Supplement: Supplementary file 3 — Description of Additional Supplementary Files [file 41467_2023_36678_MOESM3_ESM.pdf]

Description of Additional Supplementary Files

File Name: Supplementary Data 1
Description: Description of GWAS summary statistics used in LDSR.

File Name: Supplementary Data 2
Description: Description of GWAS summary statistics used in MTAG.

File Name: Supplementary Data 3
Description: Estimate of cross-trait genetic correlation of PSC among potential comorbid traits.

File Name: Supplementary Data 4
Description: MTAG-identified PSC-specific summary statistics for SNPs with P<5×10-8.

File Name: Supplementary Data 5
Description: Genomic risk loci from MTAG-identified PSC-specific GWAS using FUMA GWAS.

File Name: Supplementary Data 6
Description: Prioritized genes from MTAG PSC-specific GWAS by functional annotation and mapping of genomic risk loci of the European panels using FUMA GWAS.

File Name: Supplementary Data 7
Description: Immune-related genes prioritized by functional annotation and mapping of genomic risk loci of the European panels from MTAG PSC-specific GWAS using InnateDB.

File Name: Supplementary Data 8
Description: Description of headers for Supplementary Table 9

File Name: Supplementary Data 9
Description: Functional annotation of independent significant SNPs in genomic risk loci of the European panels from MTAG PSC-specific GWAS using FAVOR.

File Name: Supplementary Data 10
Description: Prediction of regulatory potential for MTAG-identified PSC-specific variants using MACIE.

File Name: Supplementary Data 11
Description: Fine-mapping of PSC 7 novel risk loci and 1 new independent variant at the reported risk locus using FINEMAP and GCTA-COJO.

File Name: Supplementary Data 12
Description: Colocalization results between MTAG-identified GWAS and eQTL signals (GTEx v8) calculated by coloc (PP.H4 > 0.8).

File Name: Supplementary Data 13
Description: Enrichment results amongst 406 prioritized genes using STRING protein-protein interaction networks.

File Name: Supplementary Data 14
Description: Enrichment results with FDR < 0.05 amongst 406 prioritized genes using DAVID Bioinformatics Resources.

File Name: Supplementary Data 15
Description: Pathway enrichment results for each MTAG-identified three prioritized gene (MANBA, IRF5, NKX2-3) using STRING protein-protein interaction networks.

File Name: Supplementary Data 16
Description: PSC candidate genes for drug repurposing using network-based proximity.

File Name: Supplementary Data 17
Description: Network-based proximity between drugs and PSC candidate genes for drug repurposing.

File Name: Supplementary Data 18
Description: Gene Table mapped to DrugBank Database using 406 prioritized genes by functional annotation, position, eQTL, and chromatin interaction mapping listed in Supplementary Table 6 via FUMA.
