# Supplementary material for: Multitrait genome-wide analyses identify new susceptibility loci and candidate drugs to primary sclerosing cholangitis
Source: Nat Commun. 2023 Feb 24;14:1069. doi: 10.1038/s41467-023-36678-8 (PMC9958016; doi:10.1038/s41467-023-36678-8)
Supplement: Supplementary file 22 — Reporting Summary [file 41467_2023_36678_MOESM22_ESM.pdf]

nature portfolio

Corresponding author(s): Christopher I. Amos

Last updated by author(s): 12/22/2022

Reporting Summary

Nature Portfolio wishes to improve the reproducibility of the work that we publish. This form provides structure for consistency and transparency in reporting. For further information on Nature Portfolio policies, see our Editorial Policies and the Editorial Policy Checklist.

Statistics

For all statistical analyses, confirm that the following items are present in the figure legend, table legend, main text, or Methods section.

n/a	Confirmed	
☐	☒	The exact sample size (*n*) for each experimental group/condition, given as a discrete number and unit of measurement
☐	☒	A statement on whether measurements were taken from distinct samples or whether the same sample was measured repeatedly
☐	☒	The statistical test(s) used AND whether they are one- or two-sided<br>*Only common tests should be described solely by name; describe more complex techniques in the Methods section.*
☐	☒	A description of all covariates tested
☐	☒	A description of any assumptions or corrections, such as tests of normality and adjustment for multiple comparisons
☐	☒	A full description of the statistical parameters including central tendency (e.g. means) or other basic estimates (e.g. regression coefficient) AND variation (e.g. standard deviation) or associated estimates of uncertainty (e.g. confidence intervals)
☐	☒	For null hypothesis testing, the test statistic (e.g. *F*, *t*, *r*) with confidence intervals, effect sizes, degrees of freedom and *P* value noted<br>*Give P values as exact values whenever suitable.*
☒	☐	For Bayesian analysis, information on the choice of priors and Markov chain Monte Carlo settings
☐	☒	For hierarchical and complex designs, identification of the appropriate level for tests and full reporting of outcomes
☐	☒	Estimates of effect sizes (e.g. Cohen's *d*, Pearson's *r*), indicating how they were calculated

Our web collection on statistics for biologists contains articles on many of the points above.

Software and code

Policy information about availability of computer code

Data collection	No software was used.
Data analysis	SAS v9.4: https://www.sas.com/en_us/home.html<br>R v3.6.2: https://cran.r-project.org<br>LD Score v1.0.1: https://github.com/bulik/ldsc<br>SSimp v0.5.6: https://github.com/zkutalik/ssimp_software<br>MTAG v1.0.8: https://github.com/JonJala/mtag<br>FUMA v1.4.1: https://fuma.ctglab.nl/<br>coloc v5.1.0: https://cran.r-project.org/web/packages/coloc/<br>FINEMAP v1.4.1: http://www.christianbenner.com<br>FAVOR v2.0: https://favor.genohub.org/<br>MACIE v1.0: https://github.com/ryanrsun/lungCancerMACIE/tree/master/MACIE_pipeline<br>GCTA v1.9.4: https://cnsgenomics.com/software/gcta/<br>STRING v11.5: https://string-db.org/cgi/input?sessionId=bmwWOuutn8ZR<br>DAVID Bioinformatics Resources v6.8: https://david.ncifcrf.gov/<br>LocusZoom v1.4: https://github.com/statgen/locuszoom-standalone<br>Proximity-based analysis: https://github.com/emreg00/proximity<br>Tableau Desktop Software v2022.2: https://www.tableau.com/products/desktop

For manuscripts utilizing custom algorithms or software that are central to the research but not yet described in published literature, software must be made available to editors and reviewers. We strongly encourage code deposition in a community repository (e.g. GitHub). See the Nature Portfolio guidelines for submitting code & software for further information.

Data

Policy information about availability of data

All manuscripts must include a data availability statement. This statement should provide the following information, where applicable:

- Accession codes, unique identifiers, or web links for publicly available datasets
- A description of any restrictions on data availability
- For clinical datasets or third party data, please ensure that the statement adheres to our policy

MTAG-identified PSC-specific summary statistics: https://github.com/biomedicaldatascience/PSC_MTAG
PSC GWAS summary statistics http://ftp.ebi.ac.uk/pub/databases/gwas/summary_statistics/GCST004001-GCST005000/GCST004030
The NHGRI-EBI GWAS Catalog: https://www.ebi.ac.uk/gwas/
The MRC IEU OpenGWAS database: https://gwas.mrcieu.ac.uk/
Neale's lab repository for UK Biobank GWAS summary statistics: https://github.com/Nealelab/UK_Biobank_GWAS
FinnGen repository for Finnish Biobank GWAS summary statistics r6: https://finngen.gitbook.io/documentation/v/r6/data-download
The accessible links for the GWAS summary-level data (mapped to Genome Assembly GRCh37) used in this study can be found in Supplementary Data 1 and 2.
Non-commercial DrugBank database v5.1.9: https://go.drugbank.com/releases/latest
Non-commercial DrugBank datasets can be obtained by the academic license.
eQTL data from GTEx v8: https://gtexportal.org/home/datasets and https://console.cloud.google.com/storage/browser/gtex-resources on Google Cloud Platform.
InnateDB portal: https://www.innatedb.com/redirect.do?go=resourcesGeneLists

Human research participants

Policy information about studies involving human research participants and Sex and Gender in Research.

Reporting on sex and gender	The research findings in our manuscript are based on GWAS summary statistics combining both sexes.
Population characteristics	Summary statistics from PSC GWAS samples of European ancestry were retained in this study.
Recruitment	N/A
Ethics oversight	N/A

Note that full information on the approval of the study protocol must also be provided in the manuscript.

Field-specific reporting

Please select the one below that is the best fit for your research. If you are not sure, read the appropriate sections before making your selection.

☒ Life sciences ☐ Behavioural & social sciences ☐ Ecological, evolutionary & environmental sciences

For a reference copy of the document with all sections, see nature.com/documents/nr-reporting-summary-flat.pdf

Life sciences study design

All studies must disclose on these points even when the disclosure is negative.

Sample size	We did not perform sample size calculation since we utilized the 135 existing summary-level datasets from UK Biobank, GWAS Catalog, and other previously existing GWAS in the discovery study. The range of sample size is 10,263 to 1,232,091 per trait and therefore, we believe that our findings have sufficiently statistical power.
Data exclusions	For LD score regression analysis, we restricted in HapMap 3 SNPs with MAF > 0.01 and excluded MHC region. For multi-trait joint association analysis, we selected autoimmune-related disorders with SNP-heritability greater than 0.20 and absolute value of genetic correlation between PSC and traits of interest greater than 0.2. In addition, we excluded SNPs with MAF < 0.01 and InDel/ palindromic SNPs from multi-trait joint association analysis.
Replication	Our findings have replicated 3 susceptibility loci of primary sclerosing cholangitis as detailed in the manuscript. We aggregated GWAS data of PSC (FinnGen phenocode:K11_CHOLANGI), CD (K11_CD_NOUC), UC (K11_UC_NOCD), IBD (K11_IBD), and lupus (M13_SLE) from FinnGen repository, and PBC from GWAS catalog, which are independent from those in discovery phase.
Randomization	Since the study design in our manuscript is based on an observational case-control study of a genome-wide set of genetic variants in different individuals, randomization is not applicable to the study.
Blinding	Since this is the observational case-control study, blinding is not applicable to the study.

Reporting for specific materials, systems and methods

We require information from authors about some types of materials, experimental systems and methods used in many studies. Here, indicate whether each material, system or method listed is relevant to your study. If you are not sure if a list item applies to your research, read the appropriate section before selecting a response.

Materials & experimental systems

n/a	Involved in the study
☒	☐ Antibodies
☒	☐ Eukaryotic cell lines
☒	☐ Palaeontology and archaeology
☒	☐ Animals and other organisms
☒	☐ Clinical data
☒	☐ Dual use research of concern

Methods

n/a	Involved in the study
☒	☐ ChIP-seq
☒	☐ Flow cytometry
☒	☐ MRI-based neuroimaging
